# Hypothesis driven single cell dual oscillator mathematical model of circadian rhythms

**DOI:** 10.1371/journal.pone.0177197

**Published:** 2017-05-09

**Authors:** Shiju S, K. Sriram

**Affiliations:** Center for Computational Biology, Indraprastha Institute of Information Technology-Delhi, New Delhi, India; Morehouse School of Medicine, UNITED STATES

## Abstract

Molecular mechanisms responsible for 24 h circadian oscillations, entrainment to external cues, encoding of day length and the time-of-day effects have been well studied experimentally. However, it is still debated from the molecular network point of view whether each cell in suprachiasmatic nuclei harbors two molecular oscillators, where one tracks dawn and the other tracks dusk activities. A single cell dual morning and evening oscillator was proposed by Daan et al., based on the molecular network that has two sets of similar non-redundant *per1/cry1* and *per2/cry2* circadian genes and each can independently maintain their endogenous oscillations. Understanding of dual oscillator dynamics in a single cell at molecular level may provide insight about the circadian mechanisms that encodes day length variations and its response to external zeitgebers. We present here a realistic dual oscillator model of circadian rhythms based on the series of hypotheses proposed by Daan et al., in which they conjectured that the circadian genes *per1/cry1* track dawn while *per2/cry2* tracks dusk and they together constitute the morning and evening oscillators (dual oscillator). Their hypothesis also provides explanations about the encoding of day length in terms of molecular mechanisms of *per/cry* expression. We frame a minimal mathematical model with the assumption that *per1* acts a morning oscillator and *per2* acts as an evening oscillator and to support and interpret this assumption we fit the model to the experimental data of *per1/per2* circadian temporal dynamics, phase response curves (PRC's), and entrainment phenomena under various light-dark conditions. We also capture different patterns of splitting phenomena by coupling two single cell dual oscillators with neuropeptides vasoactive intestinal polypeptide (VIP) and arginine vasopressin (AVP) as the coupling agents and provide interpretation for the occurrence of splitting in terms of ME oscillators, though they are not required to explain the morning and evening oscillators. The proposed dual oscillator model based on Daan's hypothesis supports *per1* and *per2* playing the role of morning and evening oscillators respectively and this may be the first step towards the understanding of the core molecular mechanism responsible for encoding the day length.

## Introduction

The circadian clock in the mammalian suprachiasmatic nuclei (SCN) is the master endogenous oscillator with a period close to 24 h oscillations that can persist even in the absence of external cues like light-dark (LD) cycles and temperatures. Maintaining a constant phase relationship with the external cues is important to keep the circadian clock synchronized with the local time. As a result, circadian clocks are flexible, adaptable and adjust its speed to the external zeitgeber. Alteration in circadian clock leads to neurological, metabolic, and mental disorders [1] and importantly, it is shown to adversely affect the cognitive functions like learning and memory [2]. With the advent of new biological techniques, the molecular mechanisms of gene expressions and the regulations responsible for the generation of circadian oscillations are well characterized and the time-of-day effects to the external cues are well studied in the models of fungal species Neurospora, fruit fly Drosophila, plant Arabidopsis thaliana and mammalian mice models [3–5]. Without any exceptions, circadian rhythms of all the species are tightly regulated by the interlinked multiple negative and positive feedback loops that guides circadian pacemakers to function under various conditions [6].

In mammals, the transcriptional-translation oscillator consists of positive and negative limbs with *Bmal1*, *Clock* genes and their proteins BMAL1 and CLOCK constitute the positive limbs. The CLOCK-BMAL1 heterodimer binds together to form a complex that positively regulates the negative limb genes *per1/2/3*, and *cry1/2*, and their protein products PER 1/2/3 and CRY1/2 [7] by binding to their E-box promoter regions [8]. The cytoplasmic proteins PER and CRY forms a heterodimer PER-CRY that translocates to the nucleus to repress their own transcription by binding to CLOCK-BMAL1 complex and thus establishing the negative feedback loop [9]. The orphan receptors REV-ERBα, and RORc represses and activates the transcription of *Bmal1* respectively [10, 11]. There is also a positive feedback loop by which PER2 protein regulates *Bmal1* positively [7, 12] by negatively regulating *Rev-erbα* [10] and this creates an asymmetry between *per1* and *per2* negative limbs.

One of the interesting problems that have attracted attention for a considerable period of time is to identify the molecular mechanisms responsible for encoding day length in the circadian pacemaker. It has been proposed that the encoding is done by the circadian pacemaker that consists of two distinct oscillators of which one is the morning (M) oscillator that locks on to dawn and controls the morning activities, while the other one is evening (E) oscillator that locks on to the dusk and controls the evening activities [13–15]. The concept of ME oscillators was proposed a long time back when the hamster's locomotor activity under constant light splits into two distinct components [16] and the lesion in SCN abolished the two distinct components into a single locomotor activity [17]. Even though splitting has paved way to the concept ME oscillator, its occurrence is not due to the ME oscillator [18].

Inspired by the earlier works of Pittendrigh and Daan's dual oscillator model [13], Daan et al, [15] cogently put together the criteria for morning and evening oscillators in terms of the molecular mechanisms of circadian gene expression. We define here the genes responsible for the dual oscillator in single cell are *per1/cry1* and *per2/cry2* that can oscillate and function independently, yet they influence each other. The dual oscillator is also the ME oscillator in which we assume *per1/cry1* act as M oscillator and *per2/cry2* act as an E oscillator. We provide below the summary of Daan's hypotheses (H1-H5), which we verify by building coupled set of nonlinear differential equation model based on the molecular mechanisms of circadian gene expression.

(H1) The *per1* peaks around early subjective day (CT 3–6 h) and *per2* around the late subjective day (CT 10 h) with *per1* leading *per2*. In the presence of light, *per1/cry1* phase advances (accelerates), and hence they constitute M oscillator while *per2/cry2* phase delays (decelerates) and thus constitutes the E oscillator. The peaking time of *per1* and phase advances in the presence of light indicate that *per1* may be the candidate for morning (M) oscillator, while the peaking time of *per2* during the late subjective day and light induced phase delays indicates *per2* may be the candidate for an evening (E) oscillator.

(H2) Under DD conditions, in comparison to wild type, the intrinsic period of both M and E oscillators is shorter. To translate this in terms of molecular mechanism, both *per1* and *per2* mutants under DD conditions should have a shorter period than the wild type. In the case of *per1-per2* double mutant, oscillations are not possible and therefore, ME oscillator is dysfunctional.

(H3) Circadian pacemaker exhibits bidirectional phase response curve (PRC) under light pulse perturbation with *per1* being primarily responsible for phase advances while *per2* is responsible for phase delays. In other words, suppression of phase advance is expected to occur in *per1* mutation while in *per2* mutation, suppression of phase delay is expected to occur.

(H4) Under various light-dark (LD) conditions, the phase difference between M and E oscillator increases with increase in the photoperiod with M oscillator locks onto dawn and the E oscillator locks onto the dusk.

(H5) Under constant increasing light intensity (LL conditions), the period of circadian oscillation is expected to increase in *per1* mutant mice (*per2* limb intact) while in *per2* mutant mice (*per1* limb intact), the period is expected to decrease.

In the above hypothesis, we have not included the after effects of light. Besides these five hypotheses, we also explain the occurrence of splitting phenomena through coupling of the circadian oscillator models with neuropeptides as coupling agents. In free running wheel activity, during long days, phase separation between coupled oscillators increases and at certain time, the single rhythmic activity "splits" into two different bouts with the period of each bout is less than one circadian period. We explain these splitting phenomena in terms of molecular mechanisms of circadian gene expression.

Daan et al, [15] supported their hypothesis based on experimental data obtained from both wild and mutant types mice experimental data and these data are collated from various sources that includes phase response curves (PRC) [19], phase angle differences [12, 20], and entrainment curves under different light-dark conditions [21]. Therefore, the present aim of this work is to construct a mathematical model of circadian gene regulatory network (GRN) with the assumption that *per1* and *per2* will be taken as a candidate genes for morning and evening oscillators respectively and to verify whether the dynamics of *per1/2* genes and PER1/2 proteins satisfy Daan's hypothesis H1-H5 to qualify as a dual ME oscillator in SCN.

## Materials and methods

### Mathematical model

Mathematical models for gene regulatory networks of circadian rhythms have been proposed for Neurospora [22, 23], Drosophila [23–25] and mammals [26–29] to understand the workings of circadian pacemaker under various conditions. However, till this date, there are hardly any attempts to understand the origin of ME dual oscillators from the point of view of GRN models. Previous models of ME oscillators [14, 30] are phenomenological in nature and are not based on the molecular basis of circadian oscillations and these models mostly accounted only for the splitting behavior. Existing models cannot be directly used because of various problems (see the supporting information (S1 Table) on the pros and cons of earlier models). So we develop a consensus model that is similar to most of the well known existing GRN models [26–29] but in addition, we also include detailed *per2* negative limb and add an explicit direct positive feedback loop between PER2 and *Bmal1* in the network [7, 12]. We estimate the parameters of the model from the experimental data obtained from different sources.

In developing the mathematical model for circadian rhythms to explain Daan's hypothesis for the dual oscillator model, we made the following assumptions: We do not consider (i) *cry* genes separately because Okamura et al, [20] has shown that *cry1/2* do not have distinguishable peaking rhythms as that of *per1/2* (ii) the detailed phosphorylation reactions so that the number of variables are kept to a minimum (iii) the *Rorc* gene, as it is not a part of core circadian oscillators that explains dual oscillator hypothesis (iv) the *clk* genes in the model since the CLK protein levels are constant throughout the day [31]. Finally, we consider the direct influence of light on *per1* and *per2* expression and this is different from the customary introduction of light in the model through parameters that affects the transcription rates of *per* circadian genes [26].

**Fig 1:**
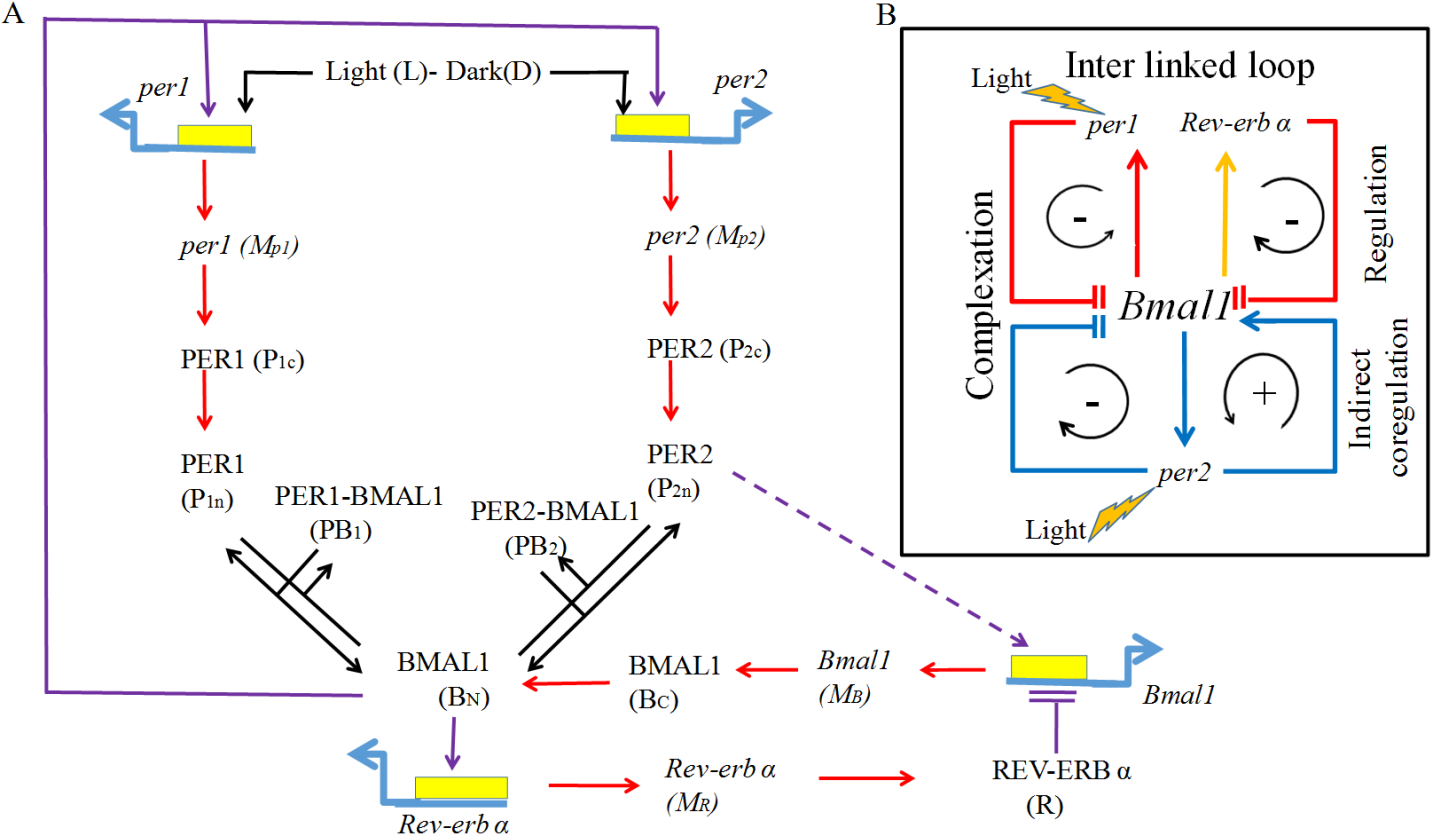
The Molecular network of circadian rhythms with feedback loops. (A) The genes and RNA's are shown in italics, while the proteins and protein complexes are shown in capitals. The dynamical variables that are used in the model are given in the brackets. Light is the external zeitgeber. The protein BMAL1 positively regulates *per1*, *per2*, and *Rev-erbα*. *per1* and *per2* produce the corresponding proteins PER1 and PER2 and these proteins interact separately with BMAL1 to form a complex PER1-BMAL1 and PER2-BMAL1 to complete the negative feedback loop. *Rev-erbα* negatively regulates *Bmal1* transcription to complete the second negative feedback loop. PER2 coregulates *Bmal1* positively [32] and closes the only indirectpositive feedback loop. (B) Summary of the complete network shown in (A) that captures three negative and one positive feedback loops.

The present mathematical model consists of (i) Hill's equation to describe the positive and negative regulations (ii) Michaelis-Menten equation to describe the degradation of mRNA's and proteins (iii) mass action kinetics to describe both the complexation and first order degradation reactions. In the model, we also consider the nuclear and cytosolic *per1/2* and *Bmal1* genes and their proteins separately. The terms that are used in the present model equations are similar to those of models [26, 28, 29], but it's different from the elaborate mass action kinetics model proposed by [27]. Therefore this can be considered as a consensus model. The variables *Mp1*, *Mp2*, *P1c*, *P1n*, *P2c*, *P2n*, *Mb*, *Bc*, *BN*, *MR*, *R*, *PB1*, and *PB2*, are *per1* mRNA, *per2* mRNA, PER1 protein (cytosol), PER1 protein (nucleus), PER2 protein (cytosol), PER2 protein (nucleus), *Bmal1* mRNA, BMAL1 protein (cytosol), BMAL1 protein (nucleus), *Rev-erbα* mRNA, REV- ERBα protein, PER1—BMAL1 complex, and PER2 -BMAL1 complex, respectively. Rates are denoted by *v*_*si*_ (*i* = 1, 2…), Michaelis constants by *k*_*ei*_'s, degradation constants by *k*_*di*_'s, production and complexation rates by *k*_*pi*_'s, m and w are Hill's coefficient to denote co-operativity, *k*_*ai*_'s denote activation constants, and *k*_*li*_ denote inhibition constant. *L* is the light parameter, which when silenced (*L* = 0), is taken to be under DD conditions. There are overall 13 equations with 60 parameters. We estimate all the parameters for the wild type by genetic algorithms to fit the mRNA's and proteins got from the experiments. We also include the positive regulation of *Bmal1* by PER2 protein that gives rise to the positive feedback loop [7,12]. This assumption is based on the experimental evidence performed invitro and invivo in NIH3T3 fibroblasts that PER2 coregulates *Bmal1* by binding with the nuclear receptor PPARα [32]. However, presently there is no evidence that this happens in SCN and we only speculate this coregulation. This speculation is added as a new feedback in the model that was not hitherto considered in the earlier models of mammalian circadian rhythms. In summary, we assume that there is an additional indirect positive feedback between *Bmal1* and PER2, where PER2 coactivates through a hitherto unknown nuclear receptor. We do not consider the indirect positive feedback of PER2 that regulates *Rev-erbα*, which in turn regulates *Bmal1* through its protein REV-ERBα in the model. The biological network with interlinked feedback loops is shown in (Fig 1).

The ODEs are

$$\frac{{dM}_{p1}}{dt}=v_{s1}\frac{B_{N}^{m}}{k_{a1}^{m}+B_{N}^{m}}-v_{1}\frac{M_{p1}}{k_{e1}+M_{p1}}-k_{d1}M_{p1}+L$$ (1)

$$\frac{{dP}_{1c}}{dt}=k_{1}M_{p1}-v_{2}\frac{P_{1c}}{k_{e2}+P_{1c}}-k_{d2}P_{1c}$$ (2)

$$\frac{{dP}_{1n}}{dt}=k_{2}P_{1c}-v_{3}\frac{P_{1n}}{k_{e3}+P_{1n}}+k_{p1}{PB}_{1}-k_{p2}P_{1n}B_{N}-k_{d3}P_{1n}$$ (3)

$$\frac{{dM}_{p2}}{dt}=v_{s2}\frac{B_{N}^{m}}{k_{a2}^{m}+B_{N}^{m}}-v_{4}\frac{M_{p2}}{k_{e4}+M_{p2}}-k_{d4}M_{p2}+L$$ (4)

$$\frac{{dP}_{2c}}{dt}=k_{3}M_{p2}-v_{5}\frac{P_{2c}}{k_{e5}+P_{2c}}-k_{d5}P_{2c}$$ (5)

$$\frac{{dP}_{2n}}{dt}=k_{4}P_{2c}-v_{6}\frac{P_{2n}}{k_{e6}+P_{2n}}+k_{p3}{PB}_{2}-k_{p4}P_{2n}B_{N}-{k_{k}P}_{1n}-k_{d6}P_{2n}$$ (6)

$$\frac{{dM}_{B}}{dt}=v_{s3}\frac{k_{I1}^{2}}{k_{I1}^{2}+R^{2}+(\frac{R}{k_{x}})}+v_{s4}\frac{P_{2n}^{w}}{k_{a3}^{w}+P_{2n}^{w}}-v_{7}\frac{M_{B}}{k_{e7}+M_{B}}-k_{d7}M_{B}$$ (7)

$$\frac{{dB}_{c}}{dt}=k_{5}M_{B}-v_{8}\frac{B_{c}}{k_{e8}+B_{c}}-k_{d8}B_{c}$$ (8)

$$\frac{{dB}_{N}}{dt}=k_{6}B_{c}-v_{9}\frac{B_{N}}{k_{e9}+B_{N}}+k_{p1}{PB}_{1}-k_{p2}P_{1n}B_{N}+k_{p3}{PB}_{2}-k_{p4}P_{2n}B_{N}-k_{d9}B_{N}$$ (9)

$$\frac{{dM}_{R}}{dt}=v_{s5}\frac{B_{N}^{s}}{k_{a4}^{s}+B_{N}^{s}}-v_{10}\frac{M_{R}}{k_{e10}+M_{R}}-k_{d10}M_{R}$$ (10)

$$\frac{dR}{dt}=k_{7}M_{R}-v_{11}\frac{R}{k_{e11}+R}-k_{d11}R$$ (11)

$$\frac{{dPB}_{1}}{dt}=-k_{p1}{PB}_{1}+k_{p2}P_{1n}B_{N}-k_{d12}{PB}_{1}$$ (12)

$$\frac{{dPB}_{2}}{dt}=-k_{p3}{PB}_{2}+k_{p4}P_{2n}B_{N}-k_{d13}{PB}_{2}$$ (13)

In the present work, CT is the circadian time with CT zero taken as the start of the subjective day under DD condition. ZT is the zeitgeber time with ZT zero taken as the start of the light phase under LD conditions. According to the convention proposed by Daan and Merrow [33], InT is the internal time given as [CT-18]_mod 24_ and ExT is the external time given as [ZT + half of the duration of the dark phase]_mod24_.

## Results

### Peaking times and phase relations of mRNA *per's*, *Bmal1*, and *Rev-erbα* under DD conditions, and the effects of light on late and early subjective night: Support of hypothesis-1 (H1)

**Fig 2:**
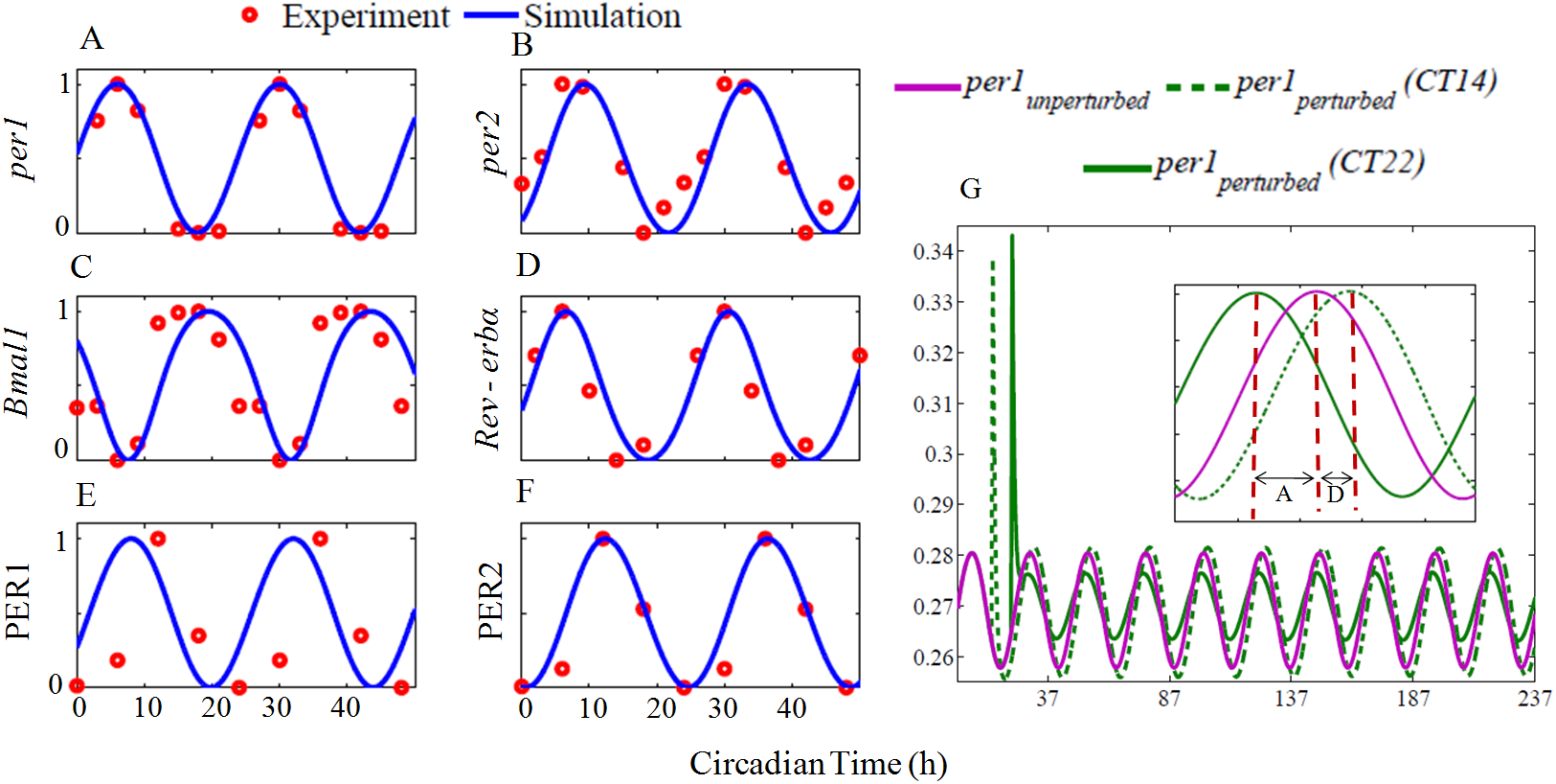
Free-running circadian oscillations under constant darkness and light pulse at certain circadian time. Blue curves are from simulation, and red circles are the experimental data points. Simulation results were obtained by integrating the model equations (Eqs 1–13) with the estimated parameters, which are given in the S2 Table (parameters used for DD, LD). For comparison, the individual time series were normalized to maximum 1 and the minimum 0. Experimental data points of *per1*, *per2*, and *Bmal1* mRNA were extracted from [7], *Rev-erbα* from [35] and PER1, PER2 protein from [34]. (G) *per* expression in response light. A 30 min light pulse with amplitude of 0.2 is applied at CT14 (early night), It shows a phase delay (green broken line). The same light pulse applied at CT 22 (late night), induce phase advance (green solid line), and agrees with the experimental results [36]. Inset shows perturbed time series came back into the original limit cycle with a delay ('D') or with an advance ('A') with respect to unperturbed time series.

Numerical simulation of the nonlinear model equations (Eqs 1–13) for the estimated parameters is shown in Fig 2A–2F. The period of free-running oscillations is 23.75 h, which is typical of mammalian circadian oscillator under constant darkness (DD) [13]. We take the light parameter *L* = 0 in the model to be the DD condition. In Table 1, we provide the simulated and experimental peaking times of all the clock components and they are in the expected range. To compare the phase relationships among the individual clock components, peaking time of *per1* mRNA at CT 6 is taken as the reference under DD conditions. The mRNA's *per1* and *per2* show a reasonable phase difference in their peaking time as seen in the experiments [7]. PER1and PER2 proteins also maintain a right phase delay from their respective *per1* and *per2* mRNA's and their peaking time are also in good agreement with the reported experimental results [34]. In summary, our model fits the data well by maintaining the right temporal phase relationships among different clock components.

**Table 1:** Comparison of different peaking time of different clock variables in CT.

Rhythmic output	Model	Experiment	Reference
*per1* mRNA	CT 6	CT 4–6	[7, 34]
*per2*mRNA	CT 9.3	CT 6–12	[7, 34]
*Rev—erb α* mRNA	CT 6.5	CT 2–6	[35,36, 37]
*Bmal1* mRNA	CT 19.4	CT 15–21	[7, 34]
PER1 protein	CT 8	CT 9–14	[7, 34]
PER2 protein	CT 12.3	CT 10–14	[7, 34]

We also simulate the effects of light at CT14 (early subjective night) and at CT22 (late night). Albrecht et al, [36] applied light pulse at CT14 and at CT22 and found to induce phase delay and advances respectively. Similarly, in simulations, the light pulse applied at early subjective night (CT14) induces phase delay while at late subjective night (CT22) induces phase advance (Fig 2G). The model simulation captures the temporal dynamics and show proper phase differences between *per1/2* mRNA's and their proteins PER1/2. Further, the model also captures phase acceleration and deceleration of light at the appropriate circadian time. This points to the fact that *per1* may be the candidate gene for M oscillator and *per2* for the E oscillator.

**Fig 3:**
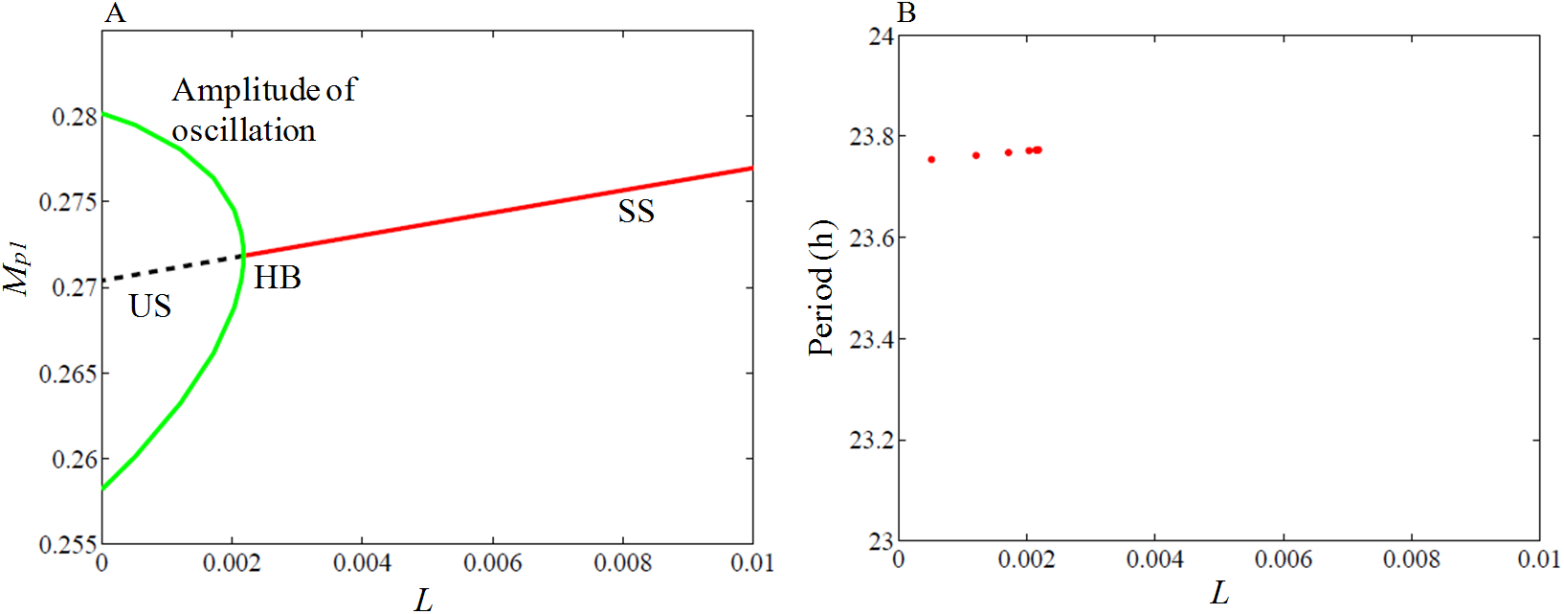
Bifurcation analysis with light *L* as the parameter. (A) Oscillation amplitude is shown in green with *L* = 0 represents the DD conditions for the variable M_p1_ (*per1* mRNA). Black broken lines are unstable steady states. As light intensity increases, sustained oscillation disappeared via supercritical Hopf bifurcation (HB) and the system enters the stable steady state (red lines). (B) Period variation as a function of light intensity. Period increases very modestly with the increase in light intensity. We used Xppaut [39] for simulating bifurcation diagrams. Simulations are obtained by integrating the model equations (Eqs 1–13) with the estimated parameters, which are given in the S2 Table (parameters used for DD, LD simulation, WT).

To consider further effects of light, we construct codimension-1 bifurcation diagram with light *L* as the bifurcation parameter (Fig 3A). For a very high value of *L*, the system remains in a stable steady state. As *L* decreases, the stable steady state becomes unstable through supercritical Hopf bifurcation, where the unstable steady state is surrounded by the stable limit cycle. Further decrease in *L* results in stable oscillations. However, the range of *L* for which the oscillations occur is very small. Unlike in many well-known models, we do not consider in our model the effects of light regulation through the parameters that affect the transcriptional production rate of *per1*, but as a stand-alone individual parameter that affects directly the rates of *per1* and *per2*. With *L* as the bifurcation parameter, we obtain an inverted supercritical Hopf bifurcation (Fig 3A). We take *L* = 0 as the DD condition that oscillates with a period of 23.75 h and is taken as the reference period. For high *L* values, as observed in many circadian experiments [13, 38], the circadian oscillations are arrhythmic and this is observed in our simulations. For the range of *L* values that we obtain oscillations, the period of the oscillator varies marginally from 23.75 to 23.8 hrs (Fig 3B).

**Fig 4:**
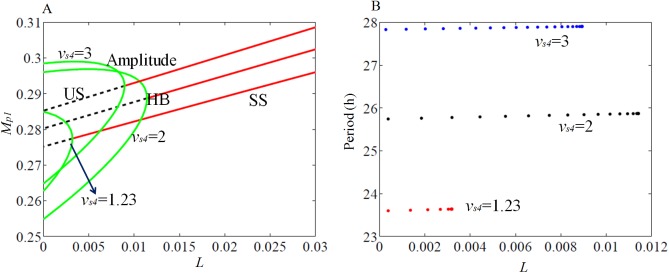
Bifurcation analysis of the effect of *Bmal1*- PER2 positive feedback loop. (A) Hopf bifurcation obtained for the range of *v*_*s4*_ that modulates the positive feedback loop. (B) The period of oscillation is shown to increase with the increase in *v*_*s4*_. *per2* mutant mice showed period decrement in LL condition [38], here we observed that the positive regulation of *Bmal1* mRNA by PER2 protein enhance the period of oscillation of the system under LL condition. Simulations are obtained by integrating the model equations (Eqs 1–13) with the estimated parameters, which are given in the S2 Table (parameters used for DD, LD simulation, WT).

We also study the effect of positive feedback between PER2 and *Bmal1* with parameter *v*_*s4*_ that contributes to the strength of feedback loop. We determine the bifurcation range and period variations with *L* as the bifurcation parameter for different *v*_*s4*_ values (Fig 4A). The increase in parameter *v*_*s4*_ increases the Hopf bifurcation regime and thereby also increases the period range with the increase of the light parameter in wild-type (Fig 4B). We also simulate the sensitivity of period to the variations of all the parameters and found that *v*_*s4*_ is highly sensitive as observed in simulations (S3 Fig).

### Dynamics of *per1* and *per2* mutants and *per1*-*per2* double mutants (Hypothesis -H2)

**Fig 5:**
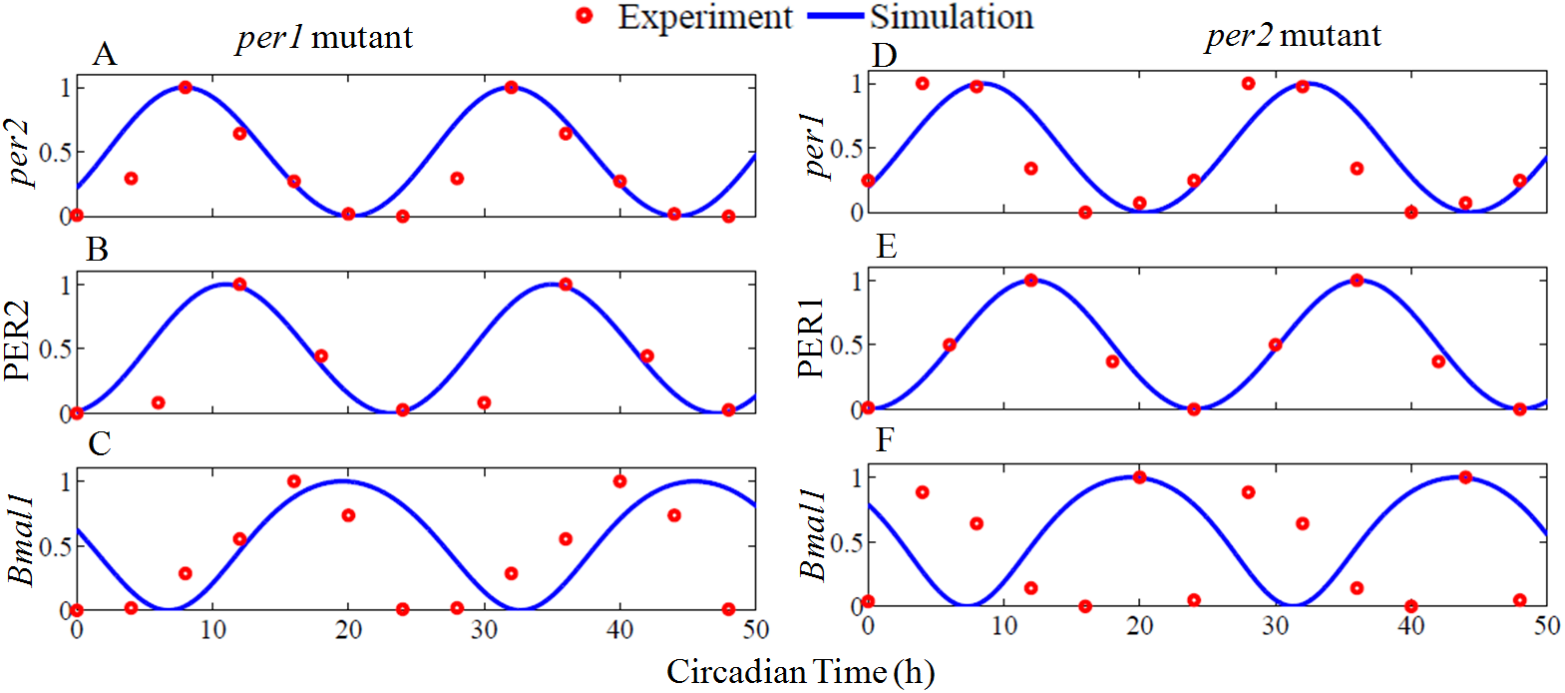
Simulation of the rhythmic behavior of *per1* and *per2* mutant mice. (A-C) are the simulations of *per1* mutant. Sustained oscillation of *per2* mRNA, PER2 protein, and *Bmal1* mRNA are shown in blue lines. The simulated results are in good agreement with the experimental data (red circle) that are observed for *per1*^ldc^ mutant mice [34]. (D-F) are the simulation shown for *per2* mutant. Similar to *per1* mutant, sustained oscillations of *per1* mRNA, PER1 protein, and *Bmal1* mRNA are observed in *per2* mutant and are in good agreement with the experimental results that observed in *per2*^ldc^ mutant mice [34]. For comparison, each time series were normalized between 0 and 1. For *per1* mutant, parameter values adjusted are *v*_*s1*_ *= 0 nMh*^*-1*^, *v*_*4*_ *= 0*.*43 nMh*^*-1*^, and *k*_*p4*_ *= 0*.*19 nMh*^*-1*^. For *per2* mutant, parameter values varied are *v*_*s1*_ *= 0*.*7 nMh*^*-1*^, *v*_*s2*_ *= 0 nMh*^*-1*^, *v*_*s3*_ *= 4*.*5 nMh*^*-1*^, *v*_*s5*_ *= 0*.*5 nMh*^*-1*^,*v*_*1*_ *= 0*.*44 nMh*^*-1*^, *v*_*2*_ *= 1*.*38 nMh*^*-1*^, *v*_*3*_ *= 1*.*67 nMh*^*-1*^,*k*_*1*_ *= 1*.*44 h*^*-1*^, *k*_*d3*_ *= 0*.*08 h*^*-1*^,*k*_*p1*_ *= 0*.*11 nM*^*-1*^*h*^*-1*^, and *k*_*p2*_ *= 0*.*18 nM*^*-1*^*h*^*-1*^. Remaining parameters are same as that of in Fig 2. *per2* mRNA that peaks at CT8 is taken as the reference point in the case of *per1* mutant, and PER1 protein that peaks at CT12 is taken as the reference point for *per2* mutant.

In experiments, under DD conditions, the period of *per1* mutant mice is shorter than the wild type [34, 38, 40], and in simulation we fit the experimental data [34] to capture this effect (Fig 5A–5C). We find that the *per1* mutant for which *per2* is functional, exhibits shorter period. In the case of *per2* mutant, the dynamics of two different mutant phenotypes, namely *per2*^*Brdm1*^ and *per2*^*ldc*^ are available in the literature and their dynamics are different. Under DD conditions, the oscillations are absent in *per2*^*Brdm1*^ mutant [12,34], whereas in the *per2*^*ldc*^ mutant, rhythmic oscillations are observed [34, 40, 41]. We simulate the behavior of both *per2*^*Brdm1*^ (S1A Fig) and *per2*^ldc^ mutants (Fig 5D–5F), but to account for hypothesis H2, we consider only *per2*^*ldc*^ mutant that exhibits oscillations. The period of the *per2*^*ldc*^ mutant in the simulation is 21.75 h and is shorter than the wild type (23.75 h), and this agrees well with the experiments [40, 34]. We also simulate *per1-per2* double mutant by silencing the transcription rates of *per1* and *per2* mRNA (*v*_*s1*_ *= 0*, *vs2 = 0*). In experiments, *per1- per2* mutant mice were found to be arrhythmic [34] and this behavior is accurately captured by our model (S1D Fig). According to hypothesis H2, this indicates that both morning and evening oscillators are dysfunctional. Besides *per1/2* knockouts, our model also correctly captures (i) the arrhythmicity in *Bmal1* mutation (S1B and S1C Fig)) as seen in the experiments in [42], (ii) a robust sustained oscillations of *Rev-erbα* as seen in the experiments [10].

In summary, our model accounts for most of the knockout phenotypes by capturing the rhythmic and the arrhythmic behaviors. In Table 2, we provide the summary of period changes seen in both simulations and experiments for wild type (WT) and the mutants. We also provide in Table 3, the peaking time of different molecular components in the mutants seen in both simulations and experiments. The shorter period in both *per1* and *per2* mutants under DD conditions and arrhythmicity in *per1-per2* double mutant indicates that the model simulations support hypothesis H2. This also indicates *per1* and *per2* as the plausible candidates for M and E oscillator respectively.

**Table 2:** Period of molecular phenotype of mutants in mammalian SCN.

Mutants	Experimental period (h)	Simulated period from model (h)	Model parameters changed from wild type	References
*per1*	21.6–23.8	23.44	*v*_*s1*_ = 0 nMh^-1^, *v*_*4*_ = 0.43 nMh^-1^, *k*_*p4*_ = 0.19 nM^-1^h^-1^	[35]
*per2*^*ldc*^*/per2*^*Brdm1*^	21.7–22.5/Arrhythmic	21.75/Arrhythmic	*v*_*s1*_ = 0.7 nMh^-1^, *v*_*s2*_ = 0 nMh^-1^, *v*_*s5*_ = 0.5 nMh^-1^, *v*_*1*_ = 0.44 nMh^-1^, *v*_*2*_ = 1.38 nMh^-1^, *v*_*3*_ = 1.67 nMh^-1^, *k*_*d3*_ = 0.08 h^-1^,*k*_*1*_ = 1.44 h^-1^, *k*_*p1*_ = 0.11nM^-1^h^-1^, *k*_*p2*_ = 0.18 nM^-1^h^-1^ (parameters for *per2*^*ldc*^ rhythmic)/ *v*_*s2*_ = 0 nMh^-1^, (for *per2*^*Brdm1*^ arrhythmic)	[12,34, 40]
*per1-per2* double mutant	Arrhythmic	Arrhythmic	*v*_*s1*_ = 0 nMh^-1^, *v*_*s2*_ = 0 nMh^-1^	[34]
*Bmal1*	Arrhythmic	Arrhythmic	*v*_*s3*_ = 0 nMh^-1^, *v*_*s4*_ = 0 nMh^-1^	[42]
*Rev-erbα*	Rhythmic	Rhythmic	*v*_*s5*_ = 0 nMh^-1^	[10]

**Table 3:** Peaking time of different oscillator components in mutants given in CT.

Mutants	Peaking time of *per1*		Peaking time of *per2*		Peaking time of *Bmal1*		Peaking time of PER1		Peaking time of PER2		Refs
	Exp	model	Exp	model	Exp	model	Exp	model	Exp	model	
*per1*	NA	NA	8	8	16	19.63	NA	NA	12	11	[34]
*per2*	5–8	8.4	NA	NA	20	19.4	12	12	NA	NA	[34]

EXP = Experiments, Refs = References, NA = Not applicable

### Phase response curves of wild and mutant types (Hypothesis-H3)

The circadian pacemaker in SCN responds differentially to light pulse at different phases of one circadian cycle. Application of light pulse during subjective day results mostly in dead zones, during early and middle of subjective night results in phase delay and during the late subjective night till the start of subjective day results in phase advance [41]. This selective bidirectional response of circadian pacemaker to light is mediated by multiple signaling pathways and it signifies that PRC depends on the time-of-day input and gated by different signaling pathways. Different scenarios on the effect of light on circadian rhythms are proposed: (i) Pittendrigh and Daan (1976) [13] postulated that the PRC of E oscillator has only phase delays while that of M oscillator exhibits only phase advances. (ii) On the other hand, Daan et al, [15] hypothesized that the PRC can be bidirectional in response to light and large phase delays will occur in the E-only oscillator, while large phase advance will occur in the M-only oscillator. These predictions are based on the observations made by Albrecht et al, [36] that in the *per1* mutant, at ZT22 light pulse suppressed phase advance, whereas in the *per2* mutant at ZT14, light pulse eliminated phase delay. (iii) Daan et al, [15] also proposed that under light pulse perturbation, phase advances of both *per1* and *per2* are suppressed during subjective day, but not during the early subjective day (see Fig 3A in [15]). Strong phase delays are possible only during the subjective night. Therefore, in this scenario, in comparison to WT, phase delays in *per1* and *per2* mutants are much larger and smaller respectively and this also encapsulates both scenarios (i) and (ii). We expect our model simulation to capture PRC of scenario (iii).

**Fig 6:**
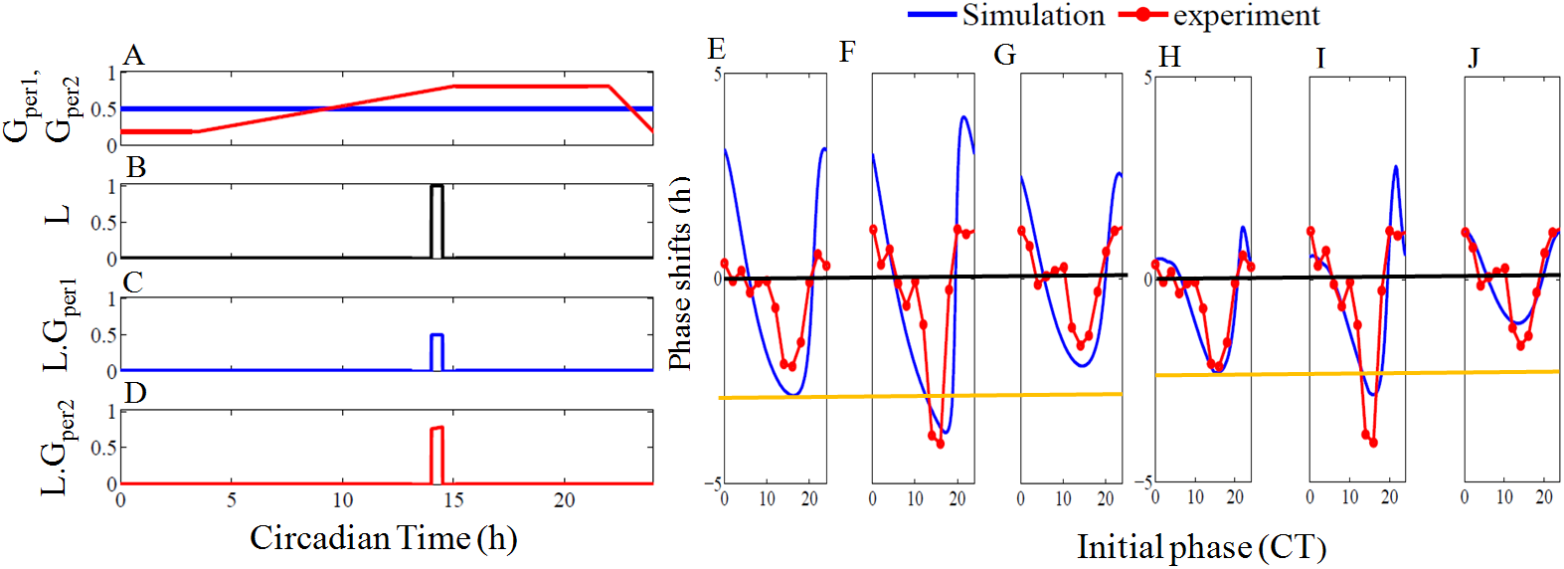
Gating variables, phase response curves of wild type, *per1*, and *per2* mutants with and without a gating variable. In the left of the figure is shown the gating variable changes the light intensity at different phases. (A) Constant and varying gating variables are used for *per1* (blue) and *per2* (red) respectively. Gating variable for *per1* is constant throughout the whole circadian period, whereas gating variable for *per2* is changed with circadian time, and reaches the maximum value between CT15 and CT20. It is at this maximum value of gating variable, a maximum delay in PRC is observed experimentally [41]. (B) An example of single light pulse with unit amplitude applied at CT14 for 30 min duration is shown. (C) The light pulse for *per1* and *per2* after multiplying with the gating variable. Light pulse for *per1* is scale down to half and the light pulse for *per2* have different values at CT14. Simulated phase response curves without the gating variable for (E) wild- type (F) *per1* mutant and (G) for *per2* mutant. Experimental data points were extracted from [41] and they are shown in red circles and a continuous line was drawn for readability. The blue lines are simulated PRC curves. To simulate PRC, light pulse *L* in the model was applied for duration of 30 mins with an amplitude value 0.35, and phase difference is measured after 10 cycles. For WT,*per1* mRNA (M_p1_) peaking at CT 6 is taken as the reference point. *per2* mRNA peaks at CT 8 is taken as the reference point when simulating *per1* mutant, and PER1 protein (P_1c_) that peaks at CT 12 is taken as the reference point when simulating *per2* mutant. For clarity, two horizontal lines are drawn; one to show the zero phases (in black) and the other magenta line in the bottom indicates the maximum phase delay of wild type. In all the experimental PRC, CT15 is the phase at which maximum delay occurs. (F) *per1* mutant PRC for which the maximum phase shift occurs at CT 15 and it is much higher than the wild type. (G) *per2* mutant PRC for which, the maximum phase shift occurs also at CT 15, but it is lower than the wild type. Simulated PRC with the gating variable for (H) wild- type (I) *per1* mutant and (J) for *per2* mutant. Compared to the PRC without a gating variable, *per2* mutant shows suppressed phase delay. Parameters for WT are as in (Fig 2), and parameters for *per1* mutants and *per2* mutant are as in (Fig 5).

To simulate scenario (iii), we construct three PRC's, one for wild type, and the other two for *per1* and *per2* mutants separately. We apply light pulse for duration of 30 min at different phases of one circadian cycle. In the case of *per2* mutant, we specifically consider *per2*^*ldc*^ mutant that oscillates under DD conditions and not the arrhythmic *per2*^*Brdm1*^ mutant. We also project the PRC simulation curves on to the PRC of the experimental data constructed from the free-running activity of mice under normal and mutant conditions [41]. The experimental PRC curves of WT and mutants exhibits phase delays during the late subjective night, advances during the early subjective day. A large phase delay is seen between CT 15–19 (Fig 6E–6G). The only fact that distinguishes PRC of mutants from wild type is that in comparison to WT, phase delays are lesser in the *per2* mutant, while in the *per1* mutant, phase delays are larger. This is also expected in Daan's hypothesis (Fig 3A in [15])

Our model simulates bidirectional PRC as seen in the experiments, but WT and *per2* mutant PRC show large phase delay than in the experiments. The maximum delay in the *per1* mutant simulation is phase shifted by 2h from the experiments. To ameliorate this problem, we introduce two gating variables, one each for the light activation of *per1* and *per2*. The idea of gating variables was previously used in the mammalian circadian models based on molecular mechanisms of gene expression [43, 44], and taking cue from there, we also similarly framed the gating expressions that act via the light variable *L* in *per1* and *per2* equations. The experimental results [45] show that photic inputs are phase gated that modifies the light responses at different phases of the oscillation. In our model, light *L* induces *per1* and *per2*, and therefore we modulate this induction through the gating variables. In order to match with experimental data, we scaled down the light parameter *L* for *per1* induction, and for *per2*, we made light parameter reach high during the early subjective night and low during both late subjective night and day. The mathematical expressions for gating variables are given below.

$$G_{per1}(t)=\frac{L_{max}}{2}$$ (14)

$$G_{per2}(t)=\left\{ \begin{array}{l} y_{1},\;mod(t,\tau )\leq t_{1}\\ \;L_{max}(\frac{1.3\;mod(t,\tau )}{\tau }),\;t_{1}>mod(t,\tau )\leq t_{2}\\ \;y_{2},\;t_{2}>mod(t,\tau )\leq t_{3}\\ \;(y_{1}-y_{2})(\frac{mod(t,\tau )-t_{3}}{t_{4}-t_{3}})+y_{2,}\;t_{3}>mod(t,\tau )\leq t_{4}\\ y_{1},\;otherwise \end{array}\right.$$ (15)

$${Where\;L}_{max}=1$$

$$t_{1}=\frac{3.5\;\tau }{24},\;t_{2}=\frac{15\;\tau }{24},\;t_{3}=\frac{22\;\tau }{24},\;t_{4}=\tau$$

$$y_{1}=L_{max}\left(\frac{1.3\;t_{1}}{\tau }\right)$$

$$y_{2}=L_{max}\left(\frac{1.3\;t_{2}}{\tau }\right)$$

In DD conditions, τ is the free-running period and in LD conditions it takes the entraining period of 24 h. The temporal dynamics of gating variables are shown in (Fig 6A). The gated PRC's are in good agreement with the experimental PRC's, but again the dead zones are not observed during subjective day (Fig 6H–6J). We modified again the gating variables and could simulate the dead zones during the subjective day besides the phase advances during the late subjective night and strong phase delays during subjective night (S2 Text, S2 Fig). These results are in agreement with Daan et al, [15] as well as Pittendrigh and Daan's [13] predictions where the dead zones, phase advances, and delays occurred at the appropriate circadian time for all the cases and we could capture the differential time-of-day effects for light pulse perturbations in the simulation. The hypothesis regarding PRC, experimental and simulated PRC results are summarized in Table 4, which strongly suggests *per1* and *per2* acts like M and E oscillator respectively.

**Table 4:** Summary of PRC.

Mutant	Hypothesis [15]	Experiment [41]	Simulation
*per1*	Suppressed advance and enhanced delay	Delay is enhanced but advance is not suppressed	Delay is enhanced but advance is not suppressed
*per2*	Suppressed delay and enhanced advance	Suppressed delay and enhanced advance	Delay is suppressed but advance is not enhanced

### Entrainment of wild type, *per*1 and *per*2 mutants to different photoperiods (Hypothesis -H4)

Entrainment of the endogenous circadian pacemaker to different external LD cycles (photoperiods) provides information about the encoding of day length. Specifically, external LD cycles of different ratios capture long or short days and endogenous circadian network should perceive and map this external cues. Therefore, circadian pacemakers should adapt and be plastic to entrain and encode variations in LD cycles. To evaluate the performance of our model, we consider different photoperiods for which we expect *per1* tracks only dawn by peaking during or close to the light phase and *per2* tracks dusk by peaking during the transition from light to dark phase.

To simulate different LD cycles, we modulate the light parameter *L* in the model as a square-wave function that goes from *L* = 0 during the dark phase to *L* = 0.1 during the light phase. Experimental results indicate that in wild-type, *per1* and *per2* mRNA levels in mammals rise up during the light phase and falls down during the dark phase [38, 46–48]. However, the peaking time of *per1* and *per2* mRNA differs significantly under different photoperiods and their phase differences increase with increase in the photo periods [38, 46].

**Fig 7:**
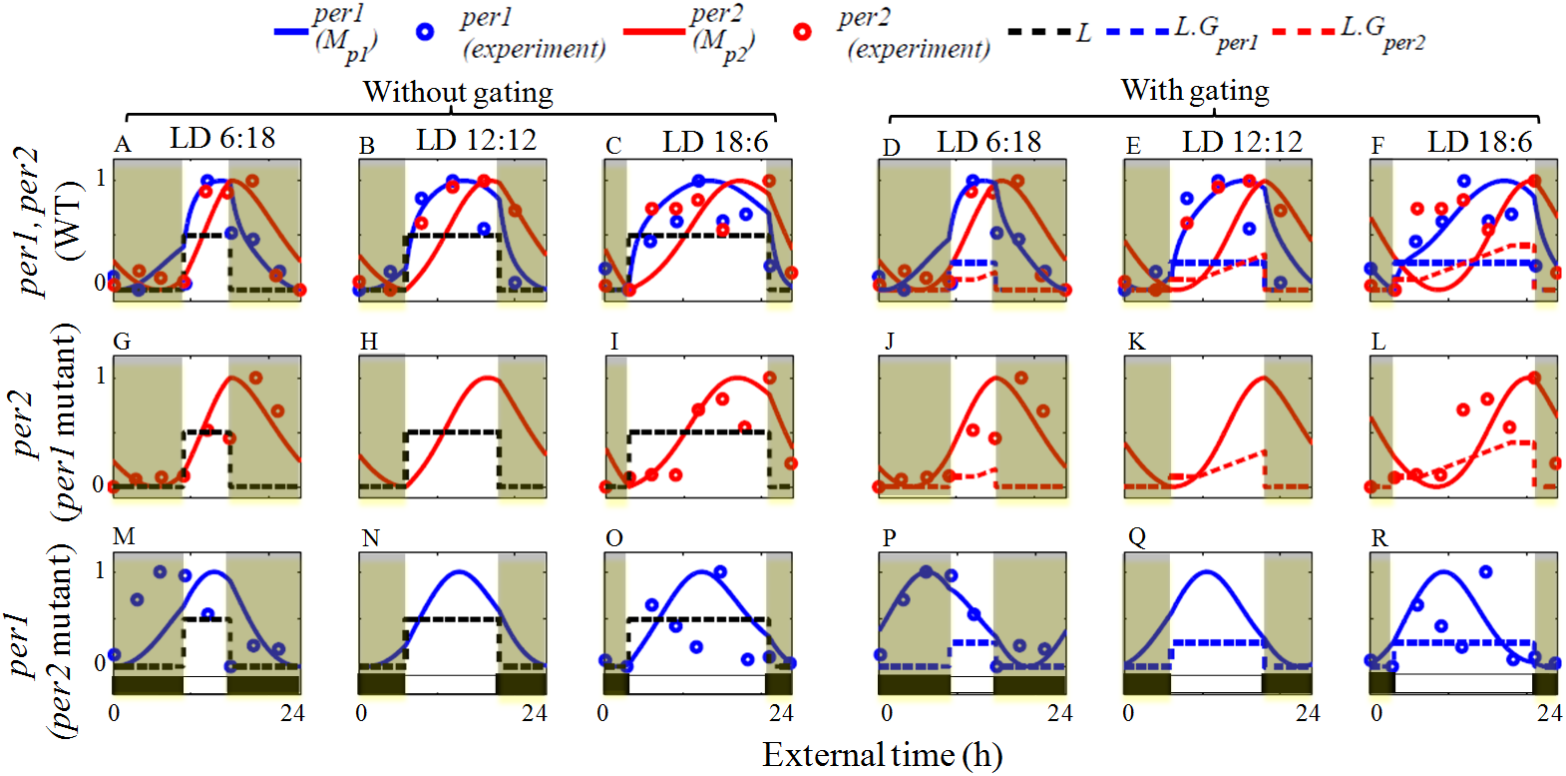
Wild type and mutant entrainment to different LD cycles with and without the gating variable. (A-C) The model entrains to various external LD cycle without the gating variable and (D-F) with gating variable (Eqs 14 and 15, Fig 6A) for WT. Blue and red lines are the simulated *per1* and *per2* mRNA, whereas blue and red circles are the experimental *per1*, *per2* mRNA respectively. Since an external cue forcing the oscillation, we consider the x axis as external time rather than circadian time. External time is defined as the middle of the light phase which in the present case is time 12. Model simulations of wild type show a maximal *per1*expression in the light phase whereas *per2* gene peaks close to dusk (off set of the light phase) during the entrainment. In (G-L) the *per1* mutant that expresses only *per2*, peaks following the dusk, and in (M-R), *per2* mutant, *per1* peak follow the dawn (on set of light phase).Under long photoperiods, *per1* mutant with gating variable displays more delay in *per2* peaking (L) than in without gating variable (I). Compared with WT, In *per2* mutant, *per1* peaks advanced both in without gating variable (M-O) and with gating variable (P-R), the magnitude of advance is more in with gating variable. Time series are normalized so that maximum value is 1 and minimum value is 0. *L* values changed in a square wave manner, during the light phase the value of *L* is 0.1 and in dark phase the value of L is 0. Experimental data points for LD 12:12 were extracted from [48], and all other data points were extracted from [38]. The dark bar in (M-R) is the dark phase, while the unfilled white bar is the light phase. This bar is also common for the entire figure and for clarity it is shown only in the last row. Parameters for WT are as in (Fig 2), and parameters for *per1* mutant and *per2* mutant are as in (Fig 5).

**Fig 8:**
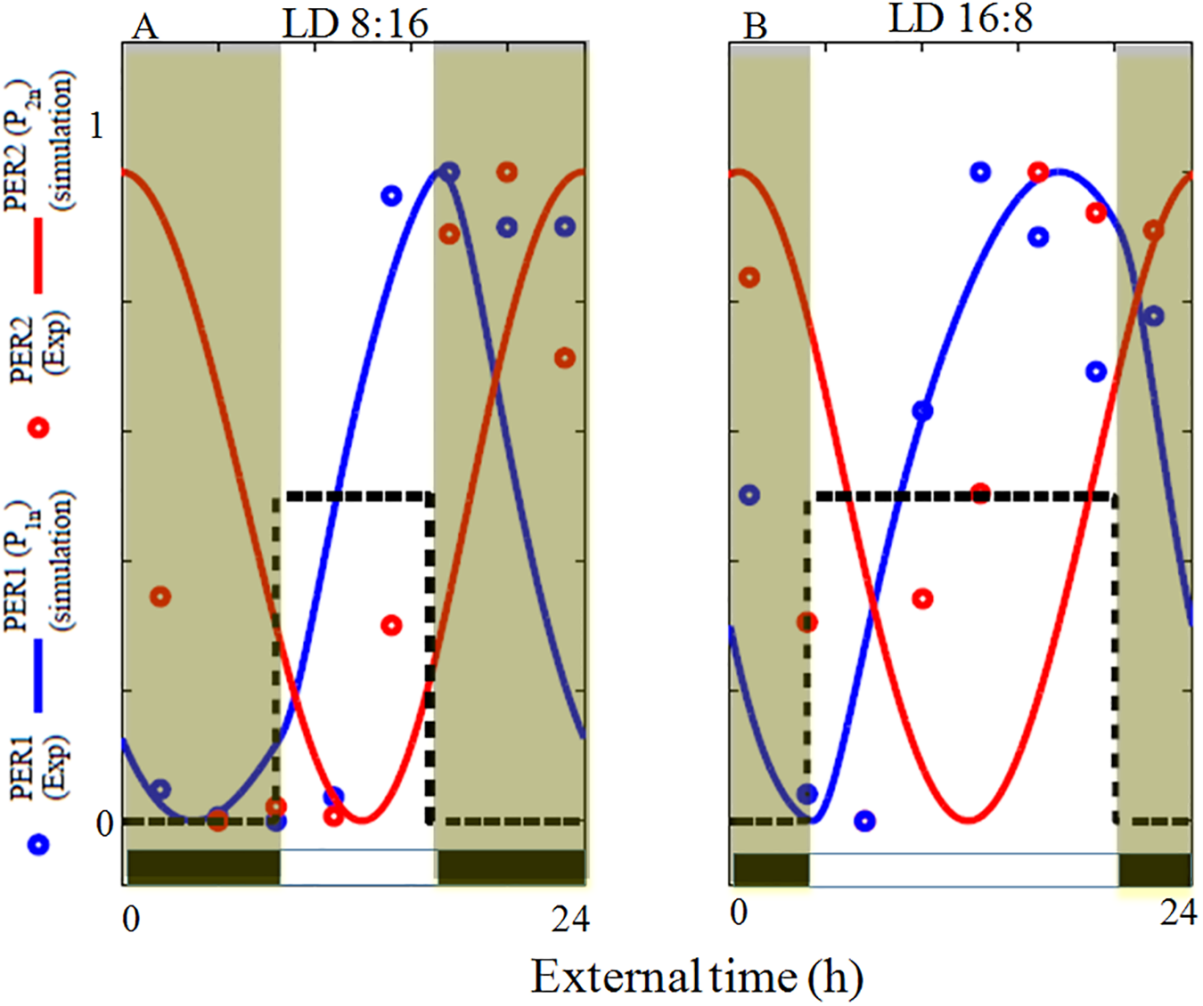
Photoperiodic variation of the PER protein. Experimental and simulated nuclear PER1 and PER2 protein under LD 8:16 (A), and LD 16:8 (B). Compared to the experimental data, phase difference between simulated PER1 and PER2 protein is higher. Experimental data points are extracted from [21]. Time series are normalized so that the maximum value is 1 and minimum value is 0.

**Fig 9:**
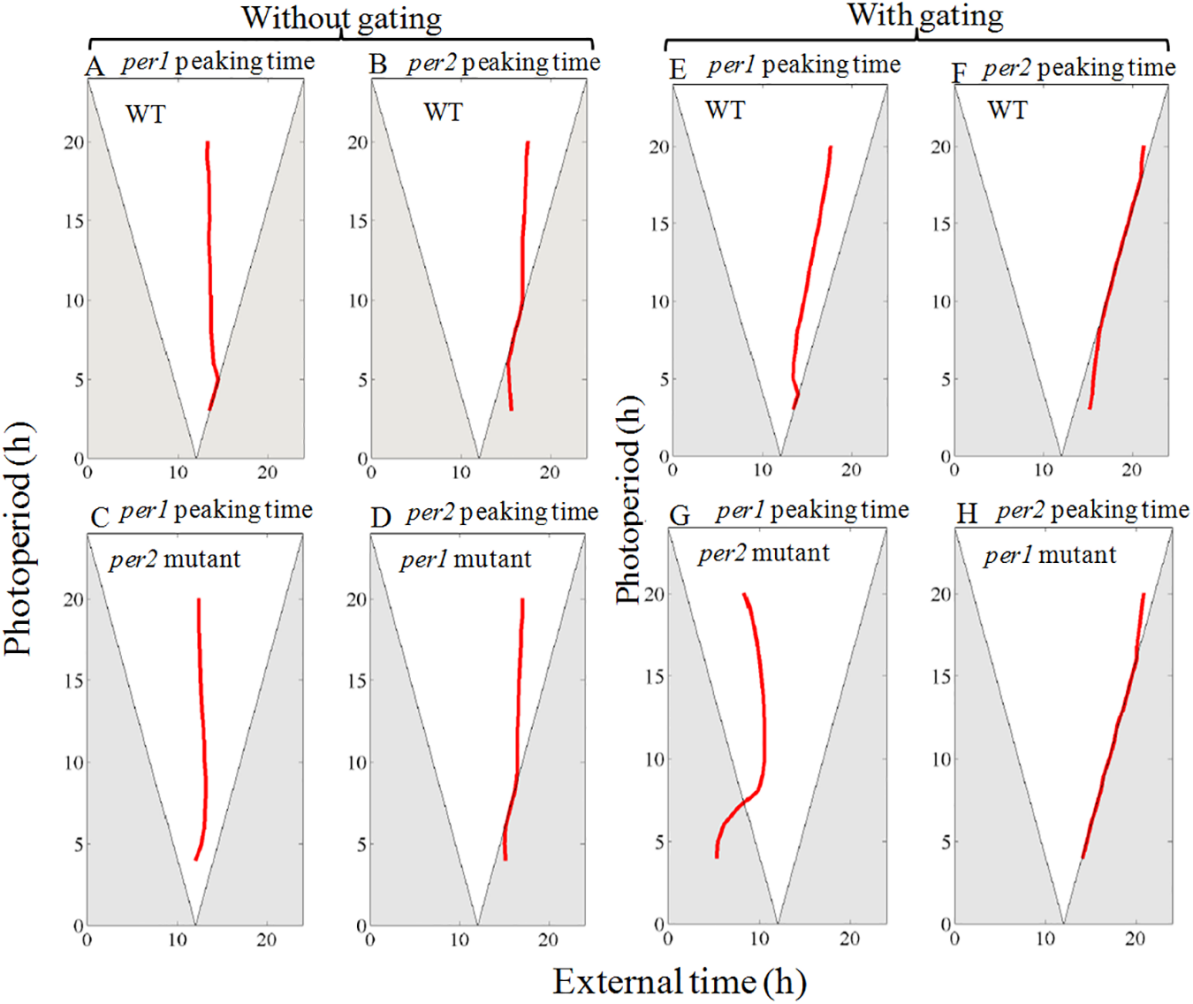
Peaking time of *per1* and *per2* mRNA under different photoperiods. (A-D) Simulation without a gating variable. (A) In WT, under short photoperiod, *per1* peaks near the dusk, photo periods increases its peaking time, shift towards the middle of the light phase. (B) *per2* peaks near the light offset, follow the dawn. (C) In *per2* mutant, *per1* always peak near the middle of the light phase. (D) In *per1* mutant, *per2* follows dusk, as in the WT. (E-H) Simulation with gating variable. (E) In WT, *per1* peaking time is shifted from middle of the light phase. (F) *per2* peaks exactly follows the dusk. (G) In *per2* mutant, *per1* peaking time always follows the dawn or late night. (H) In *per1* mutant, *per2* peaking time follows the dusk. Taking together, it is clear that *per1* is the part of M oscillator, follows the dawn and *per2* is the part of E oscillator, follows the dusk.

The simulation results of both wild and mutant types are shown in (Figs 7–9) for different photoperiods. In wild-type simulations (Fig 7A–7C), the rising portion of both *per1* and *per2* mRNA occurs during the light phase and starts to decrease during the dark phase as seen in the experiments [38, 46–48]. Under short photoperiods, *per1* peaking occurs near the light offset (Fig 7A), but as photoperiod increases, peaking occurs near the middle of the light phase (Fig 7A and 7B). On the other hand, peaking time of *per2* mRNA, as observed in the experiments [38, 46], is always close to the transition between light and dark phases (dusk) (Fig 7A–7C). We also observe that, when photoperiod increases, our model show broadening of the maxima of both *per1*, *per2* oscillations (Fig 7A–7C). In WT, under different photoperiods, the phase difference between *per1* and *per2* never went beyond 4 h (Table 5).

**Table 5:** Peaking time of *per1* and *per2* mRNA under different photoperiods.

	WT			*per1* mutant	*per2* mutant	
	*per1* peaking time (I)	*per2* peaking time (II)	Absolute phase difference between *per1* and *per2* peaking time *|I-II|*(h)	*per2* peaking time (III)	*per1* peaking time (IV)	Absolute phase difference between *per1* and *per2*peaking time *|III-IV|*(h)
DD	CT 6	CT 9.3	3.3	CT 8	CT 8.5	0.5
LD 6:18	ExT 13.84	ExT 15.26	1.42	ExT 15	ExT 13	2
LD 12:12	ExT 13.49	ExT 16.69	3.2	ExT 16.3	ExT 12.86	3.4
LD 18:6	ExT13.37	ExT 17.3	3.9	ExT 16.88	ExT 12.37	4.5
With Gating						
LD 6:18	ExT 13.52	ExT 15.83	2.3	ExT 15	ExT 6.1	8.9
LD 12:12	ExT 15.21	ExT 18	2.79	ExT 17.79	ExT 10.51	7.2
LD 18:6	ExT 17	ExT 20.77	3.77	Ex.T 20.3	ExT 9.42	10.88

ExT = external time. CT = circadian time.

To assess whether different photoperiods affects *per1* and *per2* mutants, we simulate mutants under different LD cycles. We observe that for different LD cycles, peaking time of *per2* expression in *per1* mutant show no significant variations in comparison to WT (Fig 7G–7I), and it peaks always at the transition between the light and dark phase. Similarly, for the *per2* mutant, the peaking always occurs during the light phase, but we see phase advance of *per1* gene expression in the *per2* mutant, compared with WT. We also observe an increase in the phase difference between *per1* and *per2* peaking time with the increase in photoperiod (Table 5), therefore an uncoupling of *per1* and *per2* gene expression occurred in mutants.

We also simulate the entrainment curves with gating variable that is described in the previous section. We observe that in WT with gating variable, *per1* and *per2* peaking time is delayed under long days (Fig 7D–7F) than without the gating variable (Fig 7A–7C). Peaking time of *per2* expression in the *per1* mutant (Fig 7J–7L) did not show much variations in comparison to WT (Fig 7D–7F), but the peaking time of *per1* expression in the *per2* mutant is more advanced (Fig 7P–7R).

In the case of proteins, phase difference between the peaking time of PER1 and PER2 in the Siberian hamsters [21] is very small under different LD conditions (Fig 8, red and blue circles). However, peak duration of PER protein abundance increases with increase in the photoperiod. Based on these observations, Hastings et al, [18] indicated that Daan's hypothesis of *per1*/*per2* may not be the right candidate for ME oscillators. However, we firmly believe that *per1*/*per2 mRNA's* and not their proteins PER1/PER2 may be the right candidate to look into as ME oscillators, because these proteins undergo various post-translational modifications as well as involve in diffusion process from cytoplasm to nucleus for regulation. So PER proteins may not be the right choice to explain ME oscillators. Further, Hastings et al, [18] have come to this conclusion from one case of photoperiod experiments [21]. We believe that PER protein peaking time may be different for different photoperiods and to verify this result, we simulate and plot the nuclear PER1(*P*_*1n*_) and PER2 (*P*_*2n*_) proteins time series for two different LD cycles (8:16 and 16:8) (Fig 8) and compare it with the experimental data points of Siberian hamsters [21]. Although the duration of PER1 peak increases with increase in photoperiods, our simulation results did not follow the experimental results. Besides LD cycle of 8:16 and 16:8, we also simulate for different photoperiods and found that the phase difference between PER1 and PER2 increases with increase in the photoperiods (S9 Fig). Our model simulation gives PER1 peaking time close to dusk and PER2 peaking close to midnight. There is a considerable phase difference in the peaking time of PER1 and PER2 in our simulation and this is different from what Hastings et al, [18] have proposed. Therefore, based on both mRNA and protein peaking time of *per*'s our model supports Daan's hypothesis.

We also simulate the entrainment curves for the range of photoperiod (Fig 9). *per1* peaking time in WT occur in the middle of the light phase under long photo period (Fig 9A), but for very short photoperiods our model fails to peak in the middle of light phase. In *per2* mutant, *per1* always follow the middle of the light phase (Fig 9C). In WT model, gating variable shifts the peaking time of *per1* near to light offset, however it still peaks during the day time. However, in *per2* mutant (Fig 9G), *per1* peaking time follows the light onset rather than light offset. On the other hand, peaking time of *per2* is near the light offset in both WT (Fig 9B) and in *per1* mutant (Fig 9D). By using the gating variable, peaking time of *per2* is close to dusk (Fig 9F and 9H). These simulation results clearly shows that *per1* behaves like an M oscillator, and controlled by light onset (dawn), and *per2* behaves more like an E oscillator controlled by the light offset (dusk).

### Period length variations of wild type and mutants at constant light (Hypothesis-H5)

**Fig 10:**
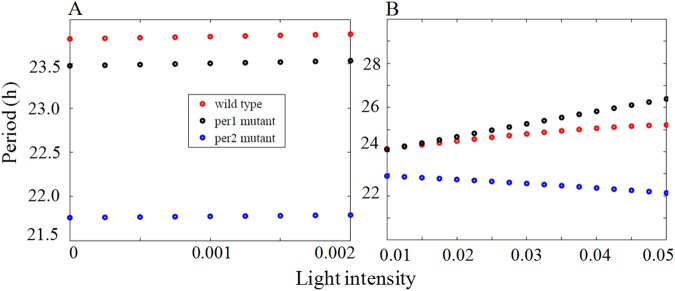
Period length as a function of light intensity. (A) Simulation results are shown for the period length variation with respect to light intensity under constant light condition for wild type (red dots), *per1* mutant (black dots) and *per2* mutants (green dots) with normal set of parameters (as in Fig 7). Oscillations are found only for the small range of values of L with the corresponding period variation also being very small. (B)) simulation results of period length variation with respect to light intensity under constant light condition with the modified set of parameters (given in the S2 Table, parameters used for LL condition). In wild type and *per1* mutant, period increases with increase in light intensity and in *per2* mutant, period decreases with increase in the light intensity, which agrees with the experimental results [38].

According to Aschoff's rule, for nocturnal animals, the period increases as a function of increasing LL, but the circadian activity decreases as a function of LL. The hypothesis is that under constant light conditions (LL) longer period is observed in the E oscillator than in the M oscillator [13, 14]. In *per1* mutant mice, where *per2* (E oscillator) is operational, it is known that the period increases with increase in light intensity [15]. In the case of *per2* mutant mice, where *per1* is operational (M oscillator), period decreases with increase in light intensity in comparison to DD [15]. The effect of light intensity on the period length under the constant light condition is shown in (Fig 10A) for the parameters that are used to fit the experimental data for DD and LD condition (as in Fig 7). The range of L values for which the period varies is very small and we didn't observe the trend seen in the experiments [38] in our simulations. To account for this effect in our model, we adjust the model parameters for both WT and mutants and the parameters are given in the S2 Table (parameters used for LL condition). Most of the parameters that we change to simulate the effects of constant light are the production and degradation rates of *Bmal1*, *per1/2*, and PER1/2. After the parameters are adjusted, the period increases with increase in light intensity in both wild-type and *per1* mutant, and this follow Aschoff's rule [49]. In the case of *per2* mutant, we observe a decrease in the period with the increase in light intensity (Fig 10B), and this did not follow the Aschoff's rule [15, 38, 50]. These results are in good agreement with the experimental results [38]. Taken together, we conclude that, under constant light condition, light accelerates *per1* and hence period decreases (frequency increases) and in the case of *per2*, light decelerates and hence the period increases (frequency decreases). This simulation supports both hypothesis H1 and H5 for the effects of light and this points to the fact that *per1* acts like an M oscillator, while *per2* acts like an E oscillator.

### Coupled oscillator model for splitting: Role of neuropeptides as coupling agents and construction of molecular actogram

**Fig 11:**
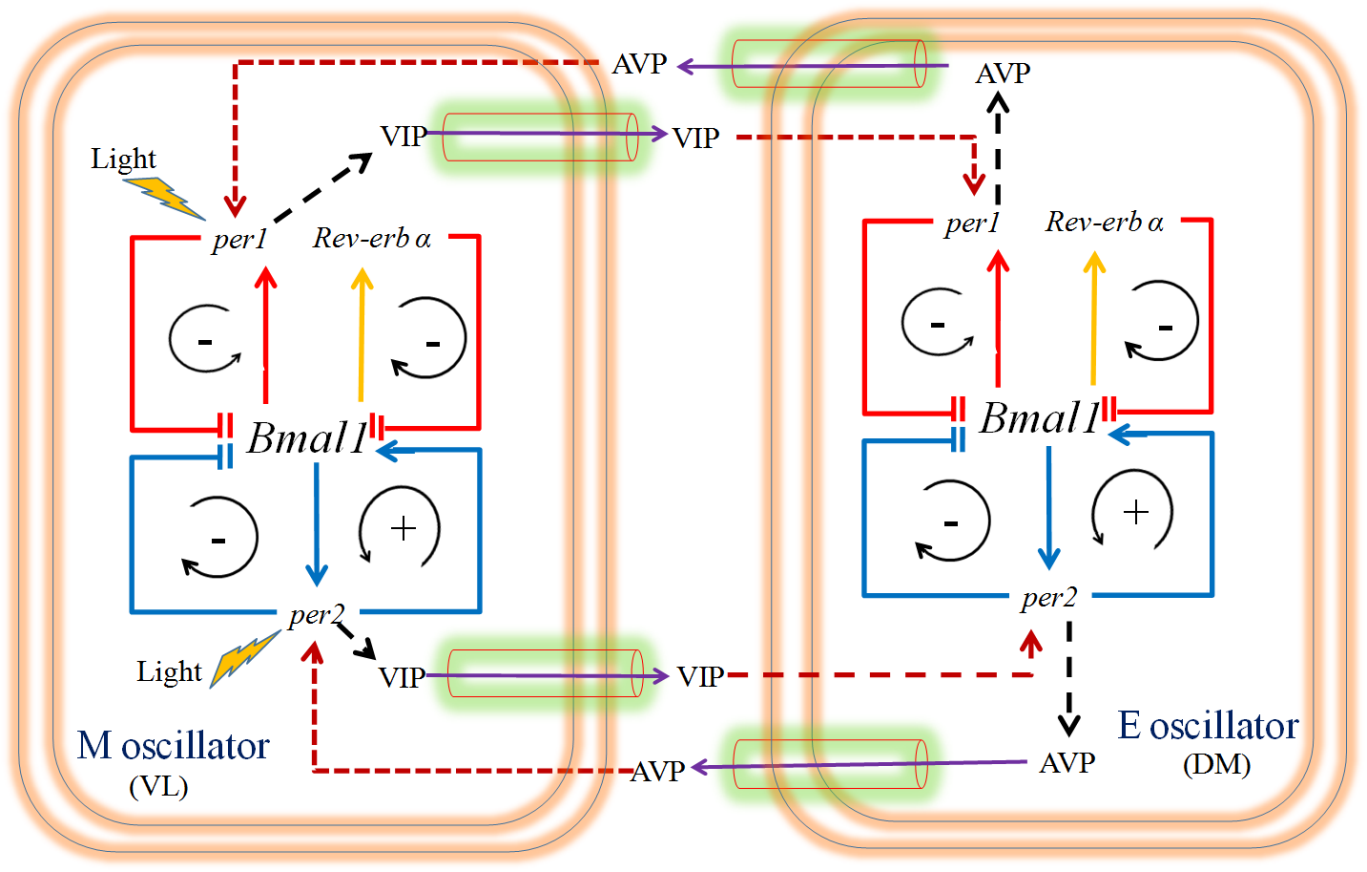
Molecular network of coupled oscillator. Coupled oscillator is constructed from two single cell dual oscillators. M oscillator is present in VL region that guides morning activities and E oscillator in DM region guides evening activities. These two oscillators respond to light differentially. Light accelerates M oscillator while light decelerates E oscillator. The two oscillators are coupled to each other by the neuropeptides AVP and VIP that acts as coupling agents for M and E oscillators. We assume here that VIP and AVP are controlled by PER1/2 in VL and DM regions respectively. Further, we assume that AVP induces *per1* and *per2* expression in the M oscillator, and VIP induces *per1* and *per2* expression in the E oscillator.

Splitting in circadian rhythms is observed under constant light when single daily bout of locomotors activity exhibits two components, one during subjective day and the other during subjective night. It has been shown in hamsters that during splitting PER1 rhythms in core and shell regions of SCN are antiphase to each other [51]. These observations of splitting in experimental data paved way to ME oscillator concept, but it's argued that to explain ME concept splitting need not be invoked (See [18] for detailed discussion). We therefore explain splitting through coupled oscillator model within our framework of dual oscillator model by making assumptions that cluster of M oscillators are present in VL region, while cluster of E oscillators are present in DM regions and they are coupled by neuropeptides that controls the phase differences between the M and E oscillators. Many coupled oscillator models [14, 30, 52–54] have been proposed to explain splitting phenomena, but they are all phenomenological in nature. For the single cell dual oscillator model that we described in earlier sections, the phase differences observed between *per1* and *per2* in WT under constant light condition (LL) was found to be very small and splitting was not seen in the simulations. Splitting appears to be a network property rather than that of a single oscillator. Therefore, taking cue from earlier works [14, 30, 52–54], we built a coupled oscillator model with neuropeptides VIP and AVP as the coupling agents. We consider in our simulation ventrolateral (VL, core) and the dorsomedial (DM, shell) regions (Fig 11) and VIP is expressed in the neurons localized mostly in the VL part of SCN [55], where light is directly received [56]. This light information is transferred to the dorsomedial part [56], where AVP neurons are located [55].VIP is arrhythmic under DD condition but displays sustained circadian oscillations under LD conditions [57]. AVP oscillates under both DD and LD conditions [58]. The network of each single cell individual oscillator has the same structure described in the earlier sections except that the parameters are different in these two oscillators. We make a strong assumption that VL region has M oscillator that guides morning activities, since it is exposed to light while DM region harbors E oscillator that is not directly exposed to light and therefore guides evening activities. For M oscillator in VL region, we choose parameters for which *per1* loop dominates based on hypothesis (H5), and here the period of the *per1* oscillator decreases with increase in light intensity [15]. Again based on the hypothesis (H5), E oscillator in DM region, we choose parameters for which *per2* loop dominates, and here the period increases with increase in light intensity [15]. Further, we assume that VIP and AVP are controlled byPER1/2 in VL and DM regions respectively and VIP induces the transcription of *per1/2* mRNA in DM region while AVP induces *per1/2* mRNA's in the VL region. The coupled oscillator model with full parameters is provided in the S4 Text and S2 Table.

**Fig 12:**
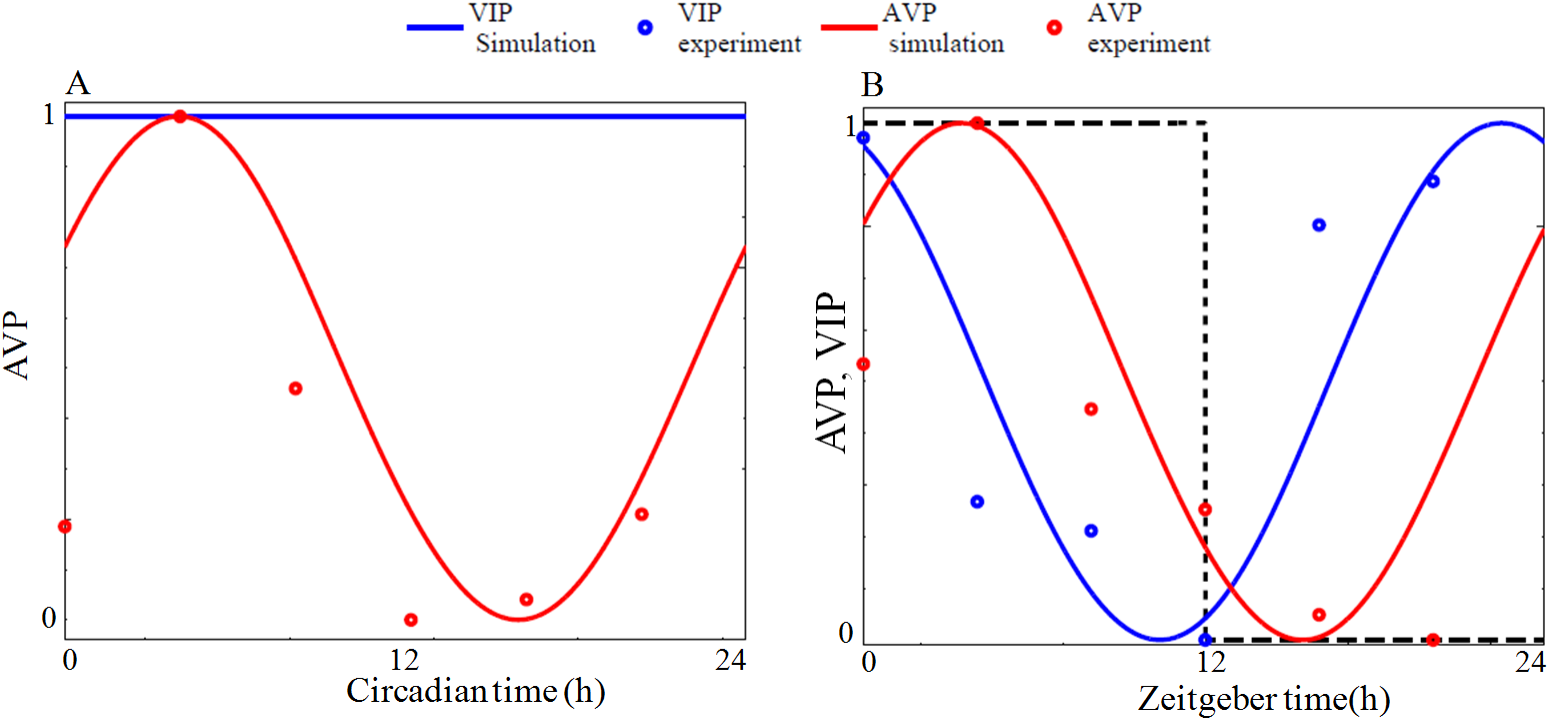
Model fitting of AVP and VIP experimental data. (A) Under DD condition only AVP shows circadian oscillation and VIP is arrhythmic. Red line indicates AVP simulation and red circle indicates AVP data from experiments [58]. (B) Under LD condition, shown in black dotted line, both VIP and AVP show sustained oscillations. Red and blue lines indicate AVP and VIP simulation respectively, while red and blue circles are the data from experiments AVP from [58] and VIP from [57] respectively. Time series are normalized with maximum and minimum as 1 and 0 respectively. Under DD condition, AVP mRNA that peaks at CT4 is taken as the reference point. Simulation results are obtained by integrating the equations given in the S4 Text (Eqs 1–28) with parameter set given in the S2 Table (ME oscillator).

To simulate splitting, we choose parameters for M (VL region) and E (DM region) oscillators that cycles with a period of 23.3 h and 23.4 h respectively under DD conditions. As it is known that period and phase of individual neurons in SCN maintain different period and phases [59], for simulation the periods of oscillators in VL and DM regions are taken differently. Light directly affects the VL region, and this light information is transferred to DM region through VIP (S4 Text Eqs 14 and 17). When VL and DM oscillators are coupled by the neuropeptides AVP and VIP, they synchronize to a common period of 23.6 h under DD conditions (Initial condition of VIP is considered as 0.1nM, and *β* = 0.01*nM*). We estimate the AVP and VIP model parameters by fitting to the experimental data of AVP and VIP [57, 58] under both DD and LD conditions (Fig 12). Like previous models [30,52], we generate different splitting patterns by varying the coupling strength under constant LL conditions with the assumption that the long exposure to light will gradually change both the coupling strengths and the expressions of both VIP and AVP neuropeptides. To construct the actogram based on molecular network simulation, we assume that PER1 protein from the VL region(*P*_*1nm*_) determines morning activities whereas the PER1 protein of the DM region(*P*_*1ne*_) determines the evening activities. We took PER1 protein in both VL and DM region to explain splitting because behaviorally split hamsters showed antiphase oscillations of PER1 expression in each side (core and shell) of the SCN [51]. It should be noted that presently there is no evidence to indicate a direct influence of *per*/PER on free running activities and this is only an assumption. To plot the actogram of molecular network model that is analogous to the actogram of free wheel running activity, we assume the normalized value of the variables *P*_*1nm*_ or *P*_*1ne*_ are above certain threshold value and these values are plotted as activities. The threshold value we define is 1.3 times the mean value of the normalized value of *P*_*1nm*_ or *P*_*1ne*_.

**Fig 13:**
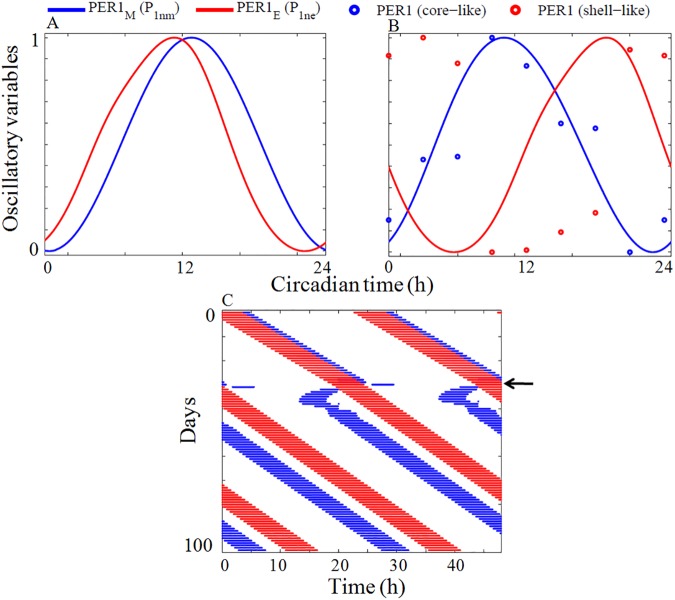
Stable split under LL condition. **(**A) The time series of PER1 protein in VL region (*P*_*1nm*_) and DM region (*P*_*1ne*_) are shown in blue and red lines respectively under normal condition. Simulation results are obtained by integrating the equations given in the S4 Text (Eqs 1–28). Initially we set VIP and AVP coupling parametersto *v*_*cm1*_ *= 0*.*35 nMh*^*-1*^, *v*_*cm2*_ *= 0*.*25 nMh*^*-1*^, and the AVP and VIP production rate to *k*_*vs1*_ *= 0*.*001 h*^*-1*^, *k*_*vs2*_ *= 0*.*015 h*^*-1*^ for unsplit condition. Rest of the parameters is given in the S2 Table (ME oscillator).The phase difference between proteins PER1, *P*_*1nm*_ of morning oscillator and *P*_*1ne*_ of the evening oscillator, is very small (~30 min). After 30 days, we vary the coupling parameters to *v*_*cm1*_ *= 0*.*28 nMh*^*-1*^, *v*_*cm2*_ *= 0*.*25 nMh*^*-1*^, and the production rates AVP and VIP to *k*_*vs1*_ *= 0*.*001h*^*-1*^, *k*_*vs2*_ *= 0*.*009 h*^*-1*^. When parameters are varied, there is an increase in phase difference between *P*_*1nm*_ (blue line) and *P*_*1ne*_ (red line). (B) Blue circles are experimentally observed PER1 protein at the core region of SCN and red circles are experimentally observed PER1 protein at the shell region of SCN, at the time of splitting [51]. (C) Simulated actogram of the coupled oscillator model. In simulations, actogram is constructed when the normalized value of the variables *P*_*1nm*_ or *P*_*1ne*_ are above certain threshold value. The threshold we define here is 1.3 times the mean value of the normalized value of *P*_*1nm*_ or *P*_*1ne*_. Blue horizontal lines represent M activities and red horizontal lines the E activities. Arrow on the right indicates the time at which parameter is varied. At the beginning of splitting, oscillator in VL region (M oscillator) has a shorter period than oscillator in DM region (E oscillator). After 30 days when parameters are varied, exchange of phase occurs between M and E components as seen in the experimental actogram (Fig 2C in [13]). Finally both the components maintained a stable phase relationship and oscillate with same period. Time series are normalized so that maximum value is 1 and minimum value is 0. We choose *L* = 0.02 for the simulation.

We reproduce many experimentally observed splitting patterns by changing the select parameters of the coupled model and one typical splitting pattern is shown in (Fig 13C) and the rest are shown in the supporting information (S7 Fig). In simulation, initially *P*_*1ne*_,the PER1 of evening oscillator (DM) phase leads over *P*_*1nm*_, the PER1 of the morning oscillator (VL) and the phase difference between PER1 of morning and evening oscillator is small (Fig 13A). After 30 days, when we decrease the coupling strength (*v*_*cm1*_) and production rate (*k*_*vs2*_) of AVP, we observe a big change in the phase difference and phase exchange takes place between *P*_*1ne*_ and *P*_*1nm*_ (Fig 13B). The model also fits the experimental data of PER1 [51] present in the core (VL) and shell (DM) regions fairly well. In experimentally observed actogram (Fig 2C in [13]), initially M and E activity components are in a fused state with E component leading over M component. After few days, when exposed to light, the two components split, and exchange of phase takes place with M component phase leads over E component. In our simulation, we capture this effect by changing the coupling parameters of the neuropeptides under constant light conditions. At the beginning of splitting, M oscillator has a shorter period than the E oscillator, after which both components maintains a stable phase relationship and oscillates with a same period. The simulated actogram (Fig 13C) is very much agrees well with the experimentally observed splitting pattern (Fig 2C in [13]).We also simulate different split and re-fused molecular actogram patterns that are observed in the free running wheel activity experiments by varying the coupling parameters and these patterns are shown in the supporting information (S7 Fig).

In summary, we have shown that the splitting phenomena can be captured by coupling two individual ME dual oscillators. We believe that the splitting is caused by ME oscillators, but these oscillators are highly regiospecific, where in one that is present in VL region with *per1*/PER1 dominant has a signature of M oscillator while the other present in DM region with *per2*/PER2 dominant has a signature of E oscillator. As a proof of concept, we coupled these oscillators through neuropeptides that modulates these oscillators and for certain coupling parameters, splitting under constant LL conditions is achieved and anti-phase relationship is maintained between the two oscillators. We also showed that different splitting patterns can be obtained when the neuropeptides are modulated and these neuropeptides are themselves induced by constant light. Therefore, through simulations, we provided a speculative molecular interpretation for splitting. However, presently, we have taken a simplistic view of taking VIP and AVP as coupling agents to generate splitting patterns, and the contribution from other neurotransmitters like GABA, NMDA, and AMPAR are yet to be studied.

## Discussion

In this work, we simulated the single cell dual oscillator model of mammalian circadian rhythms based on the hypothesis proposed by Daan et al, [15]. The hypothesis is strongly based on the collation of various experimental data that includes *per1* and *per2* peaking times, their PRC's, entrainment to different LD conditions, and importantly we also simulate the splitting behavior observed in the locomotor activities. The dual oscillator we considered in our work is that in a single cell, *per1/cry1* and *per2/cry2* are the two oscillators that can function independently, yet they influence each other. We also added a direct positive feedback from PER2 to *Bmal1* based on the experimental evidence in NIH 3T3 cells and liver tissue [32] that PER2 coregulates *Bmal1* through the nuclear receptor PPARα. However, in SCN this evidence is yet to be found. Due to lack of information, we added directly the *Bmal1* regulation by PER2 and as we get new information, we need to refine this model. We have not included the indirect regulation of PER2 on *Bmal1* through *Rev-erbα*, even though REV-ERBα regulation of *Bmal1* is included. We are particularly interested in knowing the role of *per1* and *per2* in encoding day length. In the case of splitting, the coupled oscillator model that we considered is the two single-cell oscillators that interact through neuropeptides like VIP and AVP. The important contribution of this work is that we explained the entire hypothesis H1-H5 through the construction of realistic single cell mathematical model based on the known molecular mechanisms of mammalian circadian gene and protein regulatory networks. The present single cell mathematical model does satisfy many of the Daan's hypotheses (See S1 Table for comparison of our model with four well-known models). Based on our simulations, we find that *per1* acts as an M oscillator, whereas *per2* as the E oscillator. However, our simulations are based on single cell model and have not considered the network properties of SCN that harbors more than10000 neurons on each side of SCN. We have also not considered the role of other important genes like *cry1/2* and *Rorc* in influencing *per1/2* dynamics. We have also not simulated the aftereffects of light. In spite of these drawbacks, simulations of our minimal model construct points strongly that *per1* and *per2* act as ME oscillator and encodes day length.

It's also interesting to draw a comparison between mouse and drosophila experimentalworks for ME oscillators since many of the works on dual oscillators recently has been in drosophila where clearly two distinct peaks in activity rhythm have been observed [60–63]. Helfrich-Förster et al, [60] have reviewed and compared dual oscillator model of mice and drosophila and in supporting information(S5 Table) we summarize their work and compare with our present mathematical model. Based on Helfrich-Förster et al, [60] work, it appears that presently it's too early to arrive at a consensus model of dual oscillator to describe ME activities atleast in mice and drosophila, and it's due to various factors like number of neurons and its anatomical location, differential light responses, the role of different neurotransmitters in different species, the wiring of molecular network etc. It will be interesting to model the molecular networks in drosophila for ME oscillator and compare with the mouse models for differences and similarities and the advantages/disadvantages that confer on drosophila with a fewer number of ME cells.

We also find that different splitting patterns cannot be obtained from single cell model and could simulate all the patterns only through the construction of coupled oscillator model with suitable changes in the parameters. Existing network models based on molecular interactions explain synchrony [64], and the encoding of seasonal variations [65], but these models have not separately considered the role of *per1* and *per2* in their network. It will be interesting to know whether the existing network models can explain the hypothesis if they are properly modified to include *per1* and *per2* separately. Presently it can only be speculated based on our simulations that *per1* and *per2* in a single cell act as an ME oscillator that tracks dawn and dusk respectively, but to counteract the seasonal and external variations, and noise, inter and intra cellular interactions of the population of oscillators in SCN may be necessary. In future, we intend to carry out simulations at a network level through coupling of multiple single-cell circadian oscillator models to verify Daan's hypothesis.

## Supporting information

S1 Fig Validation of the model against the arrhythmic behavior of the mutants. (A) Blue lines are the simulated *per1* mRNA for the *per2* arrhythmic mutant model (*v*_*s2*_ *= 0 nMh*^*-1*^). Red circles are the experimental data of *per1* mRNA in *per2*^*Brdm*^ mice [40]. (B) Blue lines are simulated *per1* mRNA for the *Bmal1* mutant model (*v*_*s3*_ *= 0*, *v*_*s4*_ *= 0)*. Red circles are the experimental data of *per1* mRNA in the *Bmal1*mutant mice [42]. (C) Blue lines are simulated *per2* mRNA for the *Bmal1* mutant model. Red circles are the experimental data of *per2* mRNA in *Bmal1* mutant mice [40]. (D) Blue lines are the simulated *per1* mRNA for the *per1-per2* double mutant model(*v*_*s1*_ *= 0 nMh*^*-1*^, *v*_*s2*_ *= 0 nMh*^*-1*^). Red circles are the experimental data of *per1* mRNA in the *per1-per2* double mutant mice [40]. For mutants, their transcription rate constants are made zero. Time series are normalized in such a way that maximum value is 1. Simulation results were obtained by integrating the model Eqs (1–13) in the main text. Parameters values, except transcription rate constants, are given in the S2 Table (parameters used for DD and LD).
(TIF)

S2 Fig PRC with a dead-zone. (A-C) gating variable common to both *per1* and *per2* (for more details see S2 Text). Light input is regulated by a suitable clock variable, *Bmal1* mRNA (M_B_). Simulated phase response curves with the gating for (D) wild- type (E) *per1* mutant and (F) for *per2* mutant. A dead zone is observed between CT3 to CT10 for WT, CT0 to CT10 for *per1* mutant and CT2 to CT10 for *per2* mutant. To simulate the PRC, light pulse *L* in the model was applied for a duration of 30 min with an amplitude value 0.2, and phase difference is measured after 10 cycle. The reference points for different phenotypes are similar to that of the previous PRC in the main text. Experimental data points extracted from [41] are shown in red circles and a continuous line was drawn for readability. The blue lines are simulated PRC curves from the model.
(TIF)

S3 Fig Period sensitivity of the WT-DD-model parameters. Period sensitivity of transcription rate (A), Michaelis constant (B), protein synthesis (C), enzymatic degradation (D), exponential degradation (E), activation constant (F), complex formation and dissociation (G), and Hill's coefficient (H). While considering transcription rate, *Bmal1* loop shows higher sensitivity (*v*_*s3*_, *v*_*s4*_), and *per2* loop slightly lesser (*v*_*s2*_). For all the other constants, the parameters of the *per2* loop showed higher sensitivity (*v*_*4*_, *k*_*5*_, *k*_*d4*_, *k*_*p4*_, *k*_*p3*_, *k*_*a2*_, *m*). Red arrows indicate the parameters related to the positive feedback loop between *Bmal1* and PER2 (*vs4*) which the period is shown to be highly sensitive to changes in the parameter.
(TIF)

S4 Fig Period sensitivity of the WT-LL model parameters. Period sensitivity of transcription rate (A), Michaelis constant (B), protein synthesis (C), enzymatic degradation (D), exponential degradation (E), activation constant (F), complex formation and dissociation (G), and Hill's coefficient (H). While considering transcription rate, *Bmal1* loop shows higher sensitivity (*v*_*s3*_, *v*_*s4*_), and *per2* loop slightly lesser (*v*_*s2*_). Compared with DD parameter, sensitivity of *per1* transcription rate (*v*_*s1*_) is higher than that of *per2* (*v*_*s2*_). For all the other constants, the parameters of the *per2* loop showed higher sensitivity (*v*_*4*_, *k*_*5*_, *k*_*d4*_, *k*_*p3*_, *k*_*a2*_, *m*). Red arrows indicate the parameters related to the positive feedback loop between *Bmal1* and PER2 (*vs4*) which the period is shown to be highly sensitive to changes in the parameter.
(TIF)

S5 Fig Period sensitivity of the *per1* mutant—LL model parameters. Period sensitivity of transcription rate (A), Michaelis constant (B), protein synthesis (C), enzymatic degradation (D), exponential degradation (E), activation constant (F), complex formation and dissociation (G), and Hill's coefficient (H). Here all parameters related to *per1* is completely insensitive. While considering transcription rate, *Bmal1* loop shows higher sensitivity (*v*_*s3*_, *v*_*s4*_), and *per2* loop slightly lesser (*v*_*s2*_). For all the other constants, the parameters of the *per2* loop showed higher sensitivity (*v*_*4*_, *k*_*5*_, *k*_*d4*_, *k*_*p3*_, *k*_*a2*_, *m*). Red arrows indicate the parameters related to the positive feedback loop between *Bmal1* and PER2 (*vs4*) which the period is shown to be highly sensitive to changes in the parameter.
(TIF)

S6 Fig Effect of *Bmal1*-PER2 positive feedback loop on period length variation. (A) When *v*_*s4*_ increases, period of the *per1* mutant also increase, but slope of the period variation with respect light intensity do not change much. *v*_*s4*_ for both WT and *per1* mutant increased from 0.78 to 1(B), and 1.2 (C). In both the case period should increase, but the slope of increment with respect to light intensity remain the same. Since the *per2* feedback loop is absent in *per2* mutant, it doesn't show any change in the period variation. Except *vs4*, remaining parameters are LL parameter set, that provided in the S2 Table.
(TIF)

S7 Fig Split and re-fuse under LL. (A) Simulated actogram of the model. Arrows indicate the point at which the parameter change occur. Initially set the parameters as *v*_*cm1*_ *= 0*.*35 nMh*^*-1*^,*v*_*cm2*_ *= 0*.*25 nMh*^*-1*^, *k*_*vs1*_ *= 0*.*001h*^*-1*^, *k*_*vs2*_ *= 0*.*015 h*^*-1*^ for unsplit condition, then changed the parameters (day 6-black arrow) to *v*_*cm1*_ *= 0*.*01nMh*^*-1*^, *v*_*cm2*_ *= 0*.*25 nMh*^*-1*^, *k*_*vs1*_ *= 0*.*001 h*^*-1*^, *k*_*vs2*_ *= 0*.*01 h*^*-1*^ for splitting. When splitting occurs M oscillate with a period lesser than E and finally M regains its original phase. Then we change the parameters to initial value for unsplit condition (day 44—green arrow). The simulated actogram is very much agrees well with the experimentally observed splitting pattern (Fig 2B in [13]). Here we proposed that splitting may be occur due to the transient change in the coupling strength between M and E oscillators, after the transient, the system will retains its original strength and re-fuse the splitting component. (B) Initially set the parameters as *v*_*cm1*_ *= 0*.*35 nMh*^*-1*^, *v*_*cm2*_ *= 0*.*25 nMh*^*-1*^, *k*_*vs1*_ *= 0*.*001 h*^*-1*^, *k*_*vs2*_ *= 0*.*015 h*^*-1*^ for unsplit condition, then changed the parameters (day 8-black arrow) to *v*_*cm1*_ *= 0*.*28 nMh*^*-1*^, *v*_*cm2*_ *= 0*.*25 nMh*^*-1*^, *k*_*vs1*_ *= 0*.*001h*^*-1*^, *k*_*vs2*_ *= 0*.*009 h*^*-1*^ for splitting and again change the parameter (day 41- green arrow) to *v*_*cm1*_ *= 0*.*35 nMh*^*-1*^, *v*_*cm2*_ *= 0*.*25 nMh*^*-1*^, *k*_*vs1*_ *= 0*.*004h*^*-1*^, *k*_*vs2*_ *= 0*.*025h*^*-1*^ for re-fuse. The simulated actogram is very much agrees well with the experimentally observed splitting pattern (Fig 2D in [13]).
(TIF)

S8 Fig Phase difference between P_1nm_ and P_1ne_ under parameter change. (A) The coupling term *v*_*cm1*_ changed from a lower value to higher value under LL condition (*L = 0*.*02*). It is observed that at lower values of *v*_*cm1*_, phase difference between *P*_*1nm*_ and *P*_*1ne*_ is higher, splitting will happen. However, if the coupling strength increases, phase difference decreases, unsplit condition arises. (B) The similar result observed with the parameter *k*_*vs2*_, production rate of AVP. At higher values of *k*_*vs2*_, *P*_*1nm*_ lag behind *P*_*1ne*_, and when *k*_*vs2*_ decreases, phase lead between *P*_*1ne*_ and *P*_*1nm*_ exchange and eventually splitting occurs. These results indicates that, at constant light condition some internal process take place that reduce the coupling strength between M and E oscillator, and that will lead to splitting behavior.
(TIF)

S9 Fig Peaking time of PER1 and PER2 protein under different photoperiods. PER1 peaking time in WT (A) and *per2* mutant (C) is near the light offset, as photo period increases it moves towards light phase. PER2 peaks near the midnight in both WT (B) and *per1* mutant (D). The phase difference between PER1 and PER2 increases with increase in photo period.
(TIF)

S1 Programs (ZIP)

S1 Table Comparison of new model predictions with experimental data and previous model predictions. (DOCX)

S2 Table Model parameters. The model parameters to simulate wild type and mutants under both DD and LD conditions are provided in the excel file. The file contains two sheets. In the sheet named 'single cell DD and LL', the parameters all the estimated and modified parameters are provided. In the second sheet named 'ME oscillator', we provide all the parameters that are used to simulate splitting for ME oscillators. This also includes the parameter for coupling agents VIP and AVP.
(XLSX)

S3 Table Comparison of previous model predictions on splitting. (DOCX)

S4 Table Experimental data. (XLSX)

S5 Table Comparison of ME oscillator in mammals and drosophila. (DOCX)

S1 Text Parameter estimation. (DOCX)

S2 Text modified gating variable for dead zone in PRC. (DOCX)

S3 Text Period sensitivity. (DOCX)

S4 Text Coupled oscillator model for morning and evening oscillators. (DOCX)
