# Supplementary figures and images for: Hypothesis driven single cell dual oscillator mathematical model of circadian rhythms
Source: PLoS One. 2017 May 9;12(5):e0177197. doi: 10.1371/journal.pone.0177197 (PMC5423656; doi:10.1371/journal.pone.0177197)

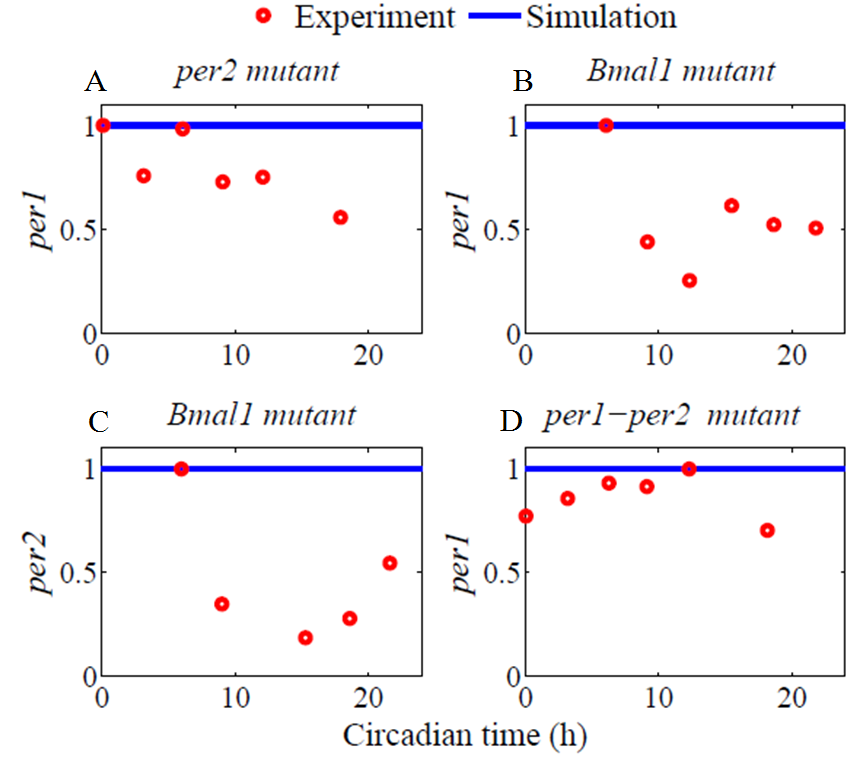

Supplement: S1 Fig — (A) Blue lines are the simulated per1 mRNA for the per2 arrhythmic mutant model (vs2 = 0 nMh-1). Red circles are the experimental data of per1 mRNA in per2Brdm mice [40]. (B) Blue lines are simulated per1 mRNA for the Bmal1 mutant model (vs3 = 0, vs4 = 0). Red circles are the experimental data of per1 mRNA in the Bmal1mutant mice [42]. (C) Blue lines are simulated per2 mRNA for the Bmal1 mutant model. Red circles are the experimental data of per2 mRNA in Bmal1 mutant mice [40]. (D) Blue lines are the simulated per1 mRNA for the per1-per2 double mutant model(vs1 = 0 nMh-1, vs2 = 0 nMh-1). Red circles are the experimental data of per1 mRNA in the per1-per2 double mutant mice [40]. For mutants, their transcription rate constants are made zero. Time series are normalized in such a way that maximum value is 1. Simulation results were obtained by integrating the model Eqs (1–13) in the main text. Parameters values, except transcription rate constants, are given in the S2 Table (parameters used for DD and LD). (TIF) [file pone.0177197.s001.tif]

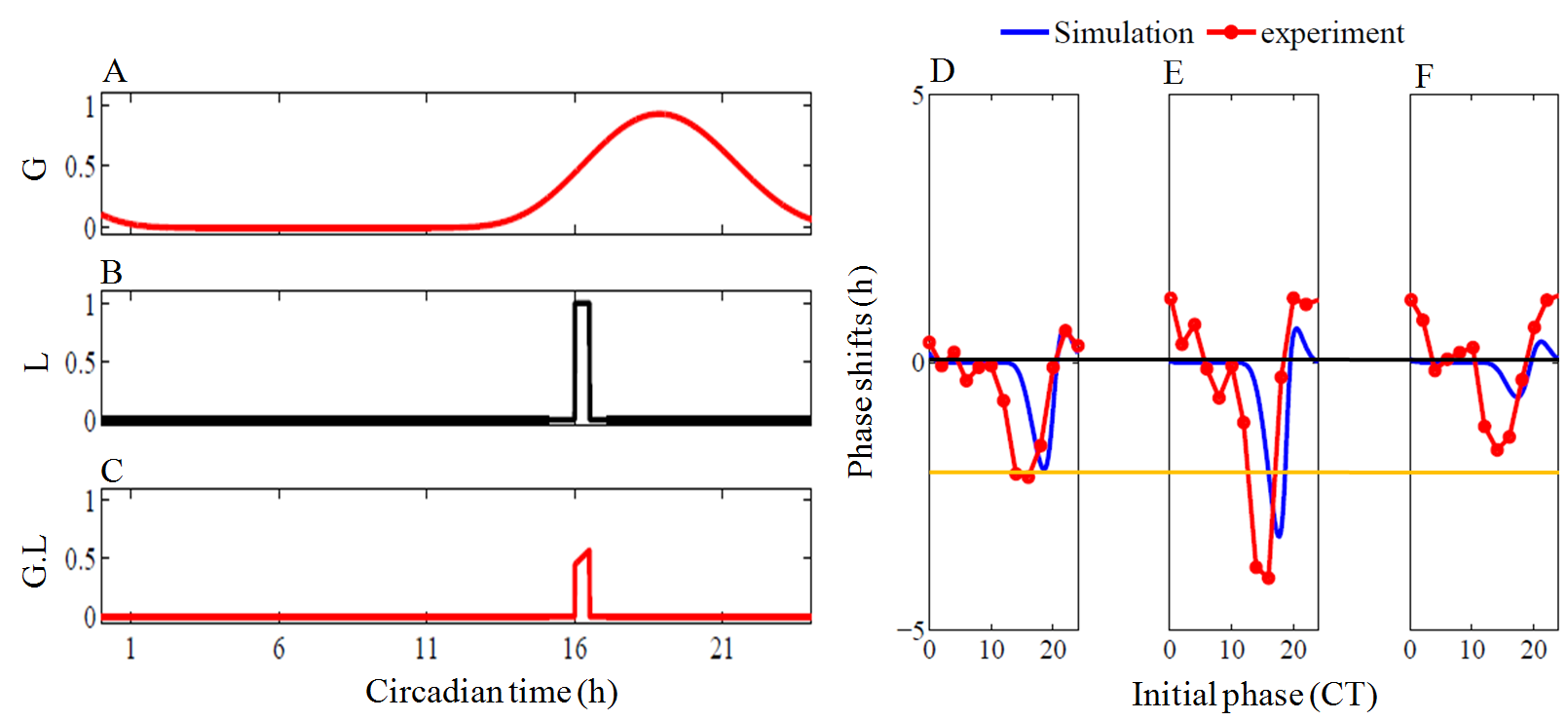

Supplement: S2 Fig — (A-C) gating variable common to both per1 and per2 (for more details see S2 Text). Light input is regulated by a suitable clock variable, Bmal1 mRNA (MB). Simulated phase response curves with the gating for (D) wild- type (E) per1 mutant and (F) for per2 mutant. A dead zone is observed between CT3 to CT10 for WT, CT0 to CT10 for per1 mutant and CT2 to CT10 for per2 mutant. To simulate the PRC, light pulse L in the model was applied for a duration of 30 min with an amplitude value 0.2, and phase difference is measured after 10 cycle. The reference points for different phenotypes are similar to that of the previous PRC in the main text. Experimental data points extracted from [41] are shown in red circles and a continuous line was drawn for readability. The blue lines are simulated PRC curves from the model. (TIF) [file pone.0177197.s002.tif]

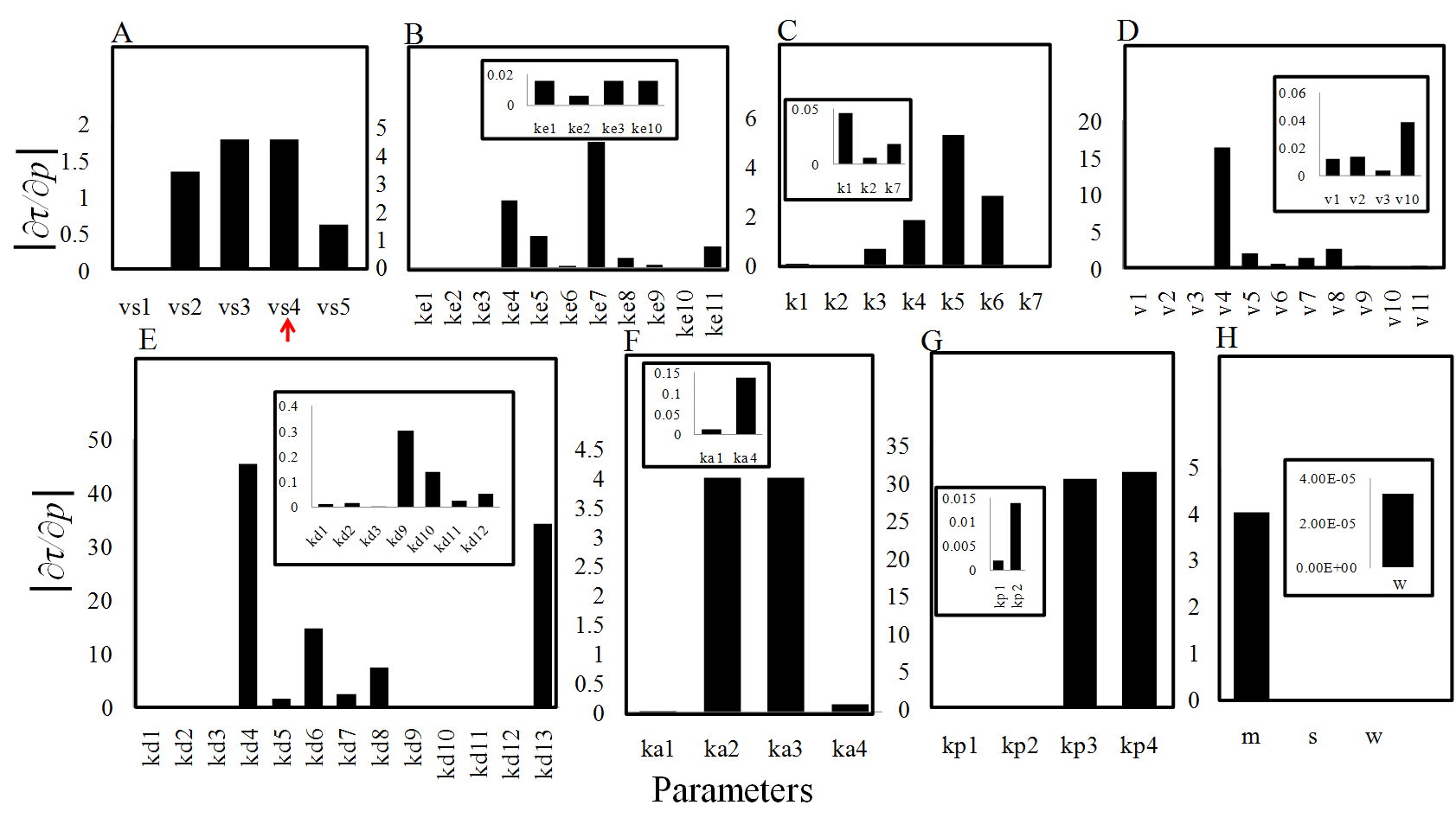

Supplement: S3 Fig — Period sensitivity of transcription rate (A), Michaelis constant (B), protein synthesis (C), enzymatic degradation (D), exponential degradation (E), activation constant (F), complex formation and dissociation (G), and Hill's coefficient (H). While considering transcription rate, Bmal1 loop shows higher sensitivity (vs3, vs4), and per2 loop slightly lesser (vs2). For all the other constants, the parameters of the per2 loop showed higher sensitivity (v4, k5, kd4, kp4, kp3, ka2, m). Red arrows indicate the parameters related to the positive feedback loop between Bmal1 and PER2 (vs4) which the period is shown to be highly sensitive to changes in the parameter. (TIF) [file pone.0177197.s003.tif]

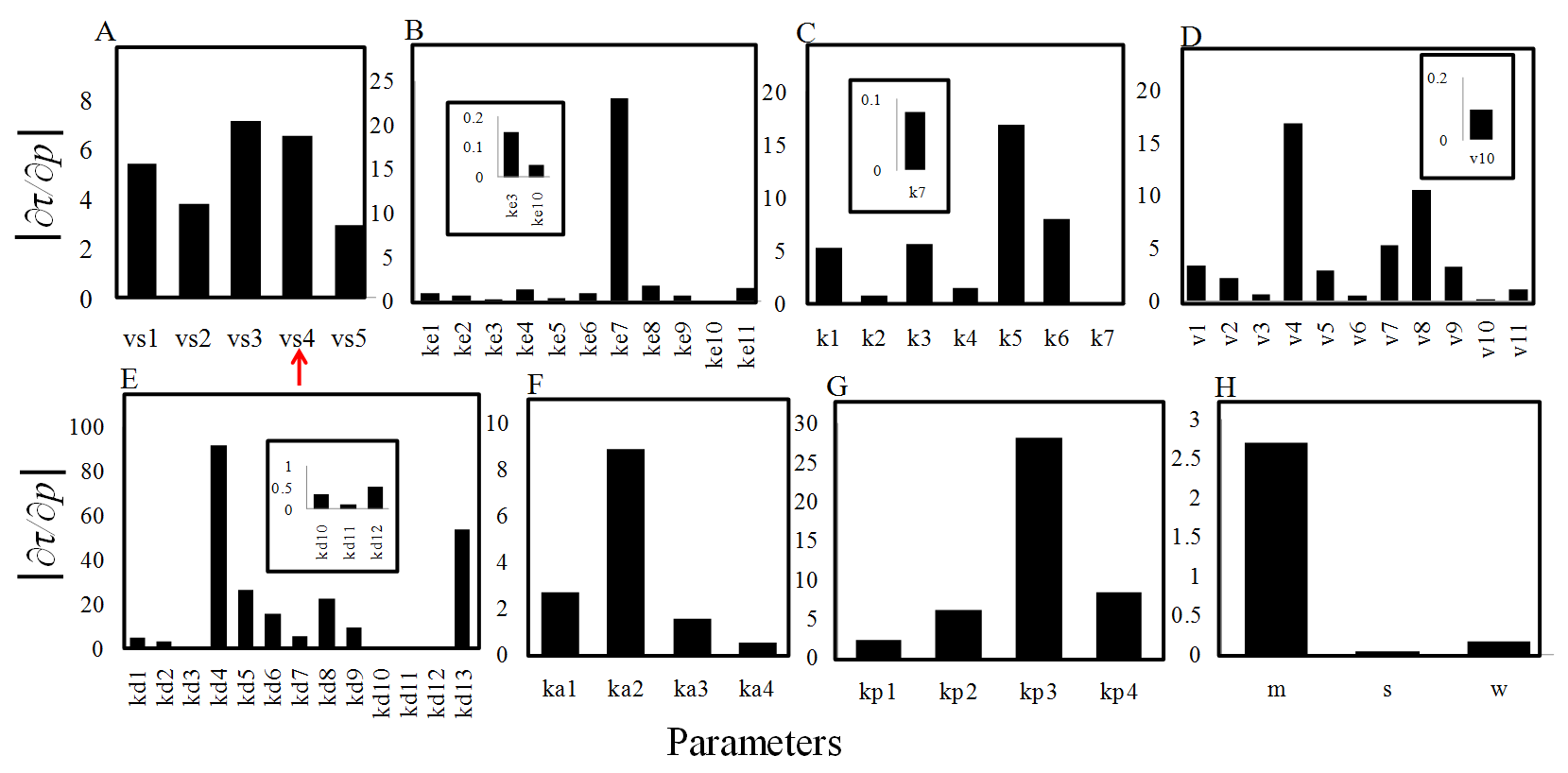

Supplement: S4 Fig — Period sensitivity of transcription rate (A), Michaelis constant (B), protein synthesis (C), enzymatic degradation (D), exponential degradation (E), activation constant (F), complex formation and dissociation (G), and Hill's coefficient (H). While considering transcription rate, Bmal1 loop shows higher sensitivity (vs3, vs4), and per2 loop slightly lesser (vs2). Compared with DD parameter, sensitivity of per1 transcription rate (vs1) is higher than that of per2 (vs2). For all the other constants, the parameters of the per2 loop showed higher sensitivity (v4, k5, kd4, kp3, ka2, m). Red arrows indicate the parameters related to the positive feedback loop between Bmal1 and PER2 (vs4) which the period is shown to be highly sensitive to changes in the parameter. (TIF) [file pone.0177197.s004.tif]

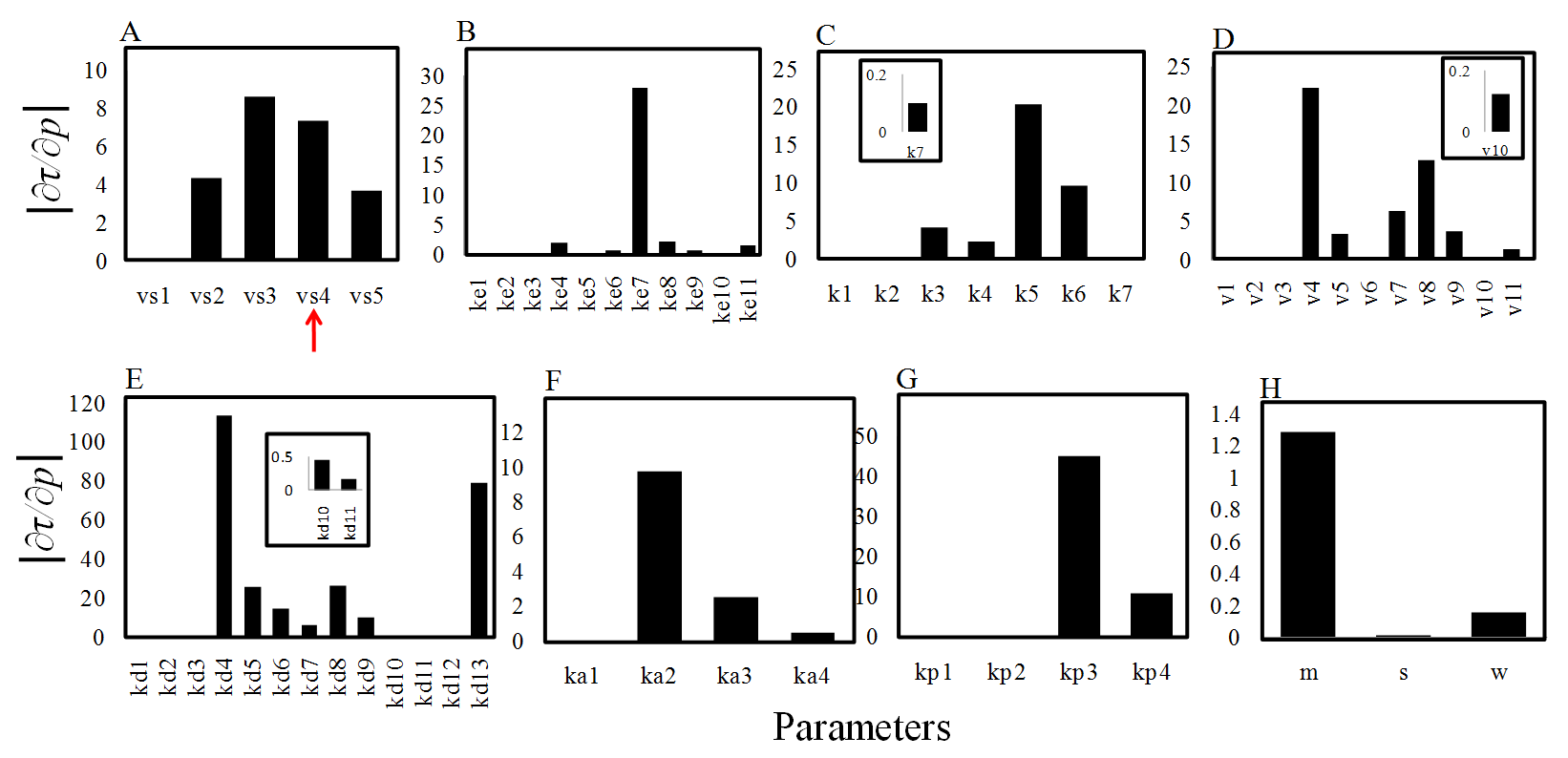

Supplement: S5 Fig — Period sensitivity of transcription rate (A), Michaelis constant (B), protein synthesis (C), enzymatic degradation (D), exponential degradation (E), activation constant (F), complex formation and dissociation (G), and Hill's coefficient (H). Here all parameters related to per1 is completely insensitive. While considering transcription rate, Bmal1 loop shows higher sensitivity (vs3, vs4), and per2 loop slightly lesser (vs2). For all the other constants, the parameters of the per2 loop showed higher sensitivity (v4, k5, kd4, kp3, ka2, m). Red arrows indicate the parameters related to the positive feedback loop between Bmal1 and PER2 (vs4) which the period is shown to be highly sensitive to changes in the parameter. (TIF) [file pone.0177197.s005.tif]

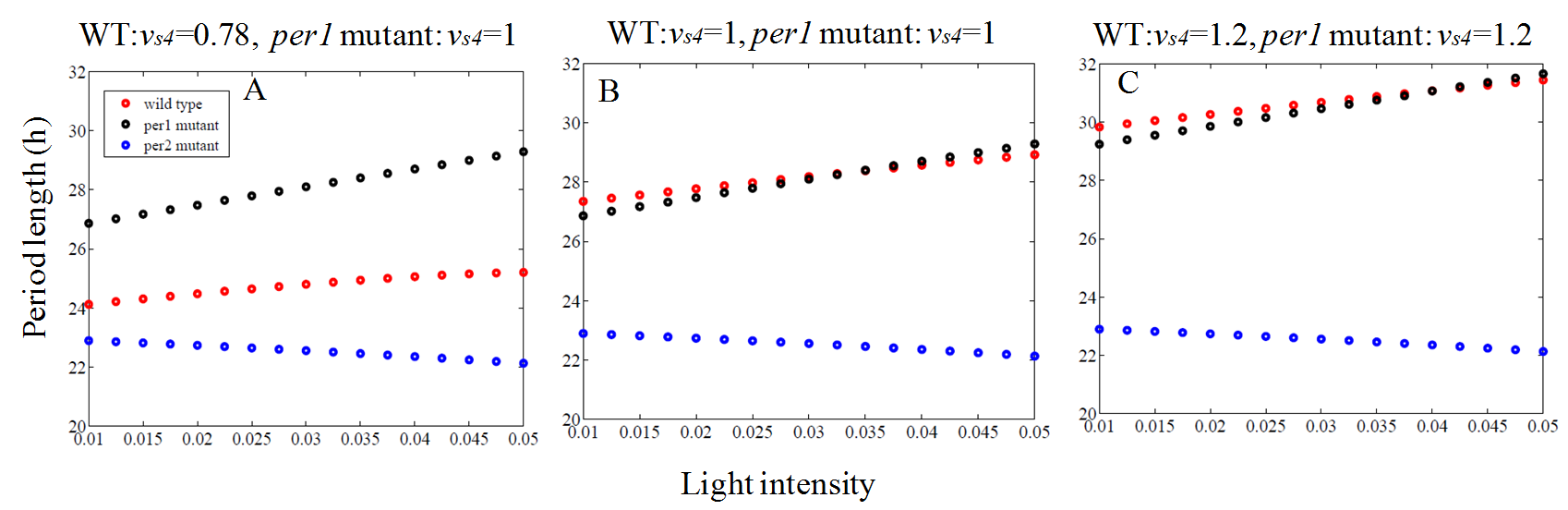

Supplement: S6 Fig — (A) When vs4 increases, period of the per1 mutant also increase, but slope of the period variation with respect light intensity do not change much. vs4 for both WT and per1 mutant increased from 0.78 to 1(B), and 1.2 (C). In both the case period should increase, but the slope of increment with respect to light intensity remain the same. Since the per2 feedback loop is absent in per2 mutant, it doesn't show any change in the period variation. Except vs4, remaining parameters are LL parameter set, that provided in the S2 Table. (TIF) [file pone.0177197.s006.tif]

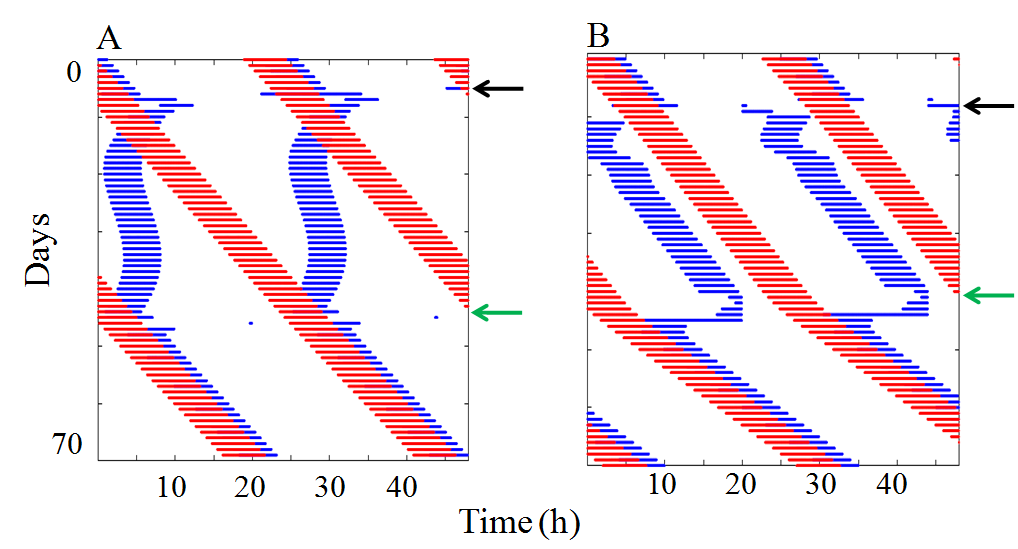

Supplement: S7 Fig — (A) Simulated actogram of the model. Arrows indicate the point at which the parameter change occur. Initially set the parameters as vcm1 = 0.35 nMh-1,vcm2 = 0.25 nMh-1, kvs1 = 0.001h-1, kvs2 = 0.015 h-1 for unsplit condition, then changed the parameters (day 6-black arrow) to vcm1 = 0.01nMh-1, vcm2 = 0.25 nMh-1, kvs1 = 0.001 h-1, kvs2 = 0.01 h-1 for splitting. When splitting occurs M oscillate with a period lesser than E and finally M regains its original phase. Then we change the parameters to initial value for unsplit condition (day 44—green arrow). The simulated actogram is very much agrees well with the experimentally observed splitting pattern (Fig 2B in [13]). Here we proposed that splitting may be occur due to the transient change in the coupling strength between M and E oscillators, after the transient, the system will retains its original strength and re-fuse the splitting component. (B) Initially set the parameters as vcm1 = 0.35 nMh-1, vcm2 = 0.25 nMh-1, kvs1 = 0.001 h-1, kvs2 = 0.015 h-1 for unsplit condition, then changed the parameters (day 8-black arrow) to vcm1 = 0.28 nMh-1, vcm2 = 0.25 nMh-1, kvs1 = 0.001h-1, kvs2 = 0.009 h-1 for splitting and again change the parameter (day 41- green arrow) to vcm1 = 0.35 nMh-1, vcm2 = 0.25 nMh-1, kvs1 = 0.004h-1, kvs2 = 0.025h-1 for re-fuse. The simulated actogram is very much agrees well with the experimentally observed splitting pattern (Fig 2D in [13]). (TIF) [file pone.0177197.s007.tif]

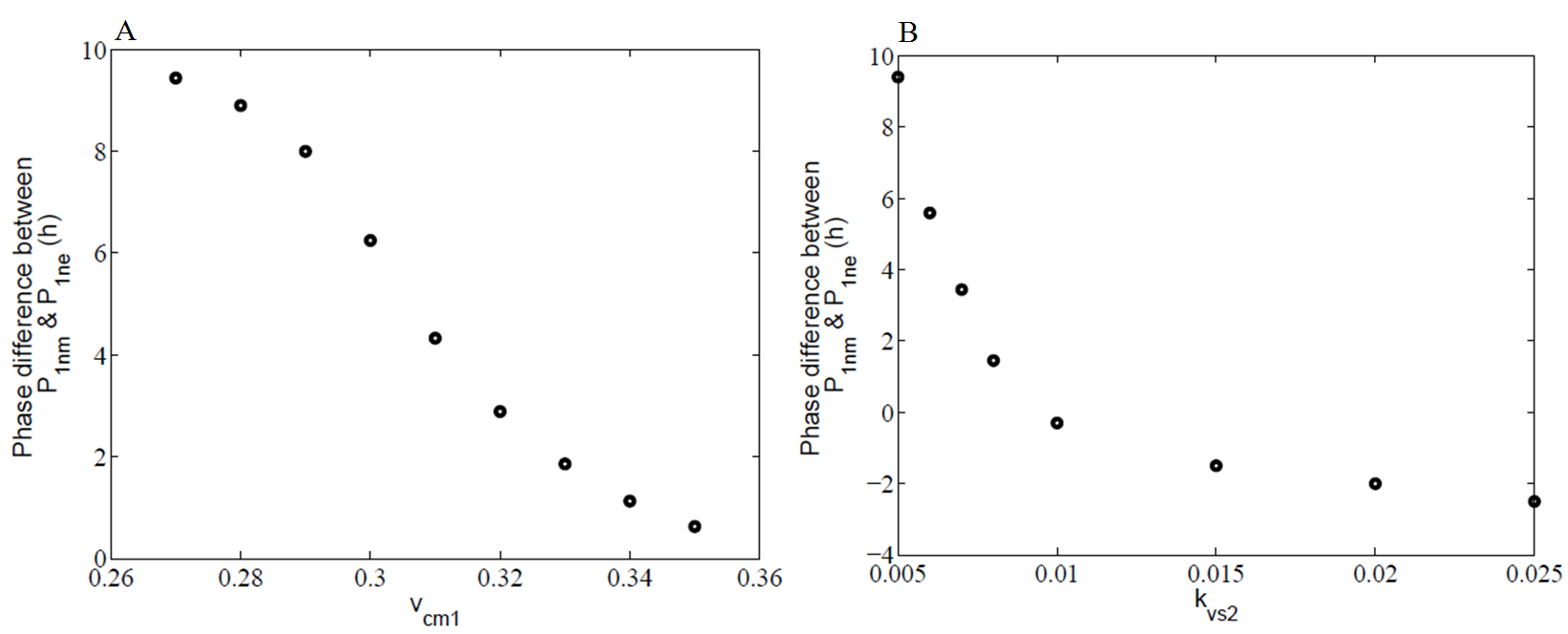

Supplement: S8 Fig — (A) The coupling term vcm1 changed from a lower value to higher value under LL condition (L = 0.02). It is observed that at lower values of vcm1, phase difference between P1nm and P1ne is higher, splitting will happen. However, if the coupling strength increases, phase difference decreases, unsplit condition arises. (B) The similar result observed with the parameter kvs2, production rate of AVP. At higher values of kvs2, P1nm lag behind P1ne, and when kvs2 decreases, phase lead between P1ne and P1nm exchange and eventually splitting occurs. These results indicates that, at constant light condition some internal process take place that reduce the coupling strength between M and E oscillator, and that will lead to splitting behavior. (TIF) [file pone.0177197.s008.tif]

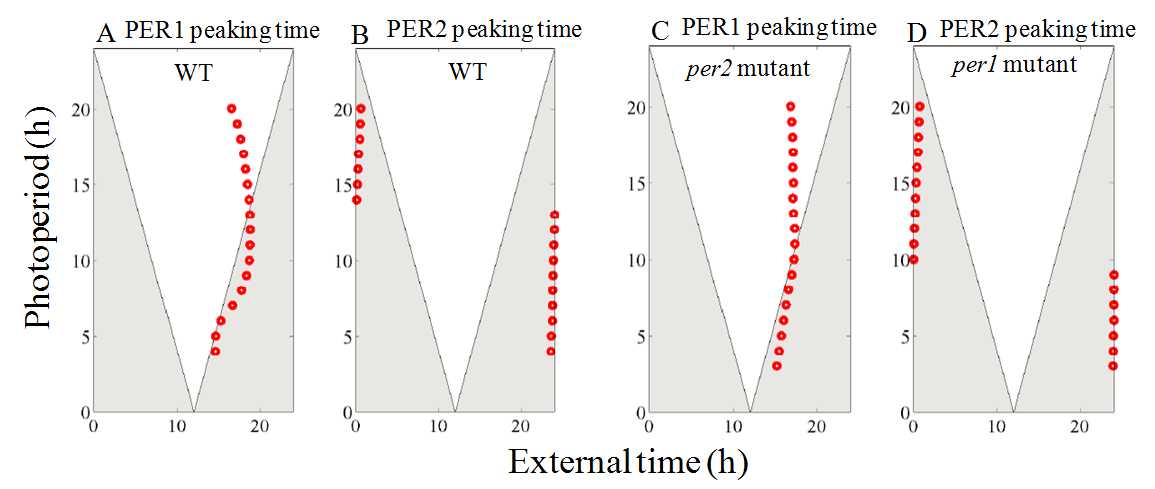

Supplement: S9 Fig — PER1 peaking time in WT (A) and per2 mutant (C) is near the light offset, as photo period increases it moves towards light phase. PER2 peaks near the midnight in both WT (B) and per1 mutant (D). The phase difference between PER1 and PER2 increases with increase in photo period. (TIF) [file pone.0177197.s009.tif]
