# Supplementary material for: Hypothesis driven single cell dual oscillator mathematical model of circadian rhythms
Source: PLoS One. 2017 May 9;12(5):e0177197. doi: 10.1371/journal.pone.0177197 (PMC5423656; doi:10.1371/journal.pone.0177197)
Supplement: S1 Table — (DOCX) [file pone.0177197.s011.docx]

**S1 Table. Comparison of New model predictions with experimental data and previous model predictions.**

| ME oscillator characteristics | | | Experiments | Forger and Peskin, 2003  [1] | Mirsky et al., 2009  [2] | Becker-Weimann et al., 2004  [3] | Leloup and Goldbeter  2003  [4] | New model |
| --- | --- | --- | --- | --- | --- | --- | --- | --- |
| Number of Variable / parameter | | |  | 73/36 | 21/132 | 7/24 | 16/55 | 13/60 |
| Period length Under DD | Wild type | | < 24 | < 24 | < 24 | < 24 | < 24 | < 24 |
|  | *per1* mutant | | < 24 | >24^*^ | Arrhythmic | NA | Arrhythmic | < 24 |
|  | *per2* mutant | | Arrhythmic | Arrhythmic | Arrhythmic | Arrhythmic | NA | Arrhythmic/<24 |
| Period length Under LL | Wild type | | >24 | < 24^*^ | NA | < 24^*^ | > 24^*^ | >24 |
|  | *per1* mutant | | >24 | < 24* | NA | NA | Arrhythmic | >24 |
|  | *per2* mutant | | <24/  Arrhythmic | Arrhythmic* | NA | Arrhythmic | NA | <24/ Arrhythmic |
| Period length variation with increasing light intensity in LL(Aschoff"s rule) | Wild | | Period increases | Period decreases^*^ | NA | Period decreases^*^ | Period increases^*^ | Period increases |
|  | *per1* mutant | | Period increases | Period decreases^*^ | NA | NA | Arrhythmic^*^ | Period increases |
|  | *per2* mutant | | Period decreases | Arrhythmic^*^ | NA | Arrhythmic^*^ | NA | Period decreases |
| Entrainment | Wild | *per1*  peaking time | Light phase | Light phase^*^ | NA | NA | Light phase | Light phase |
|  |  | *per2*  peaking time | Closer to dusk | Light phase^*^ | NA | Light phase^*^ | NA | Closer to dusk |
|  | *per1* mutant | *per2*  peaking time | Closer to dusk | Light phase ^*^ | NA | NA | NA | Closer to dusk |
|  | *per2* mutant | *per1*  peaking time | Light phase | Arrhythmic^*^ | NA | Arrhythmic^*^ | NA | Light phase |
| PRC  (Whether it matched with experimental results or not) | Without gating variable | |  | Not matched | NA | Not  matched | Matched | Matched |
|  | With gating variable^+^ | |  | Matched | NA | Matched | NA | Matched |

* Results were not shown in the literature, but we obtained from simulation.

^+^ Different Gating variables were used for different models.

NA: Not applicable

**References**

1. Forger DB, Peskin CS. A detailed predictive model of the mammalian circadian clock. Proc Natl Acad.Sci U S A. 2003 Dec 9;100(25):14806-11.
2. Mirsky HP, Liu AC, Welsh DK, Kay SA, Doyle FJ. A model of the cell-autonomous mammalian circadian clock. Proc Natl Acad Sci U S A. 2009 Jul 7;106(27):11107-12.
3. Becker-Weimann S, Wolf J, Herzel H, Kramer A. Modeling feedback loops of the mammalian circadian oscillator. Biophys J*.* 2004 Nov 30;87(5):3023-34.
4. Leloup JC, Goldbeter A. Toward a detailed computational model for the mammalian circadian clock. Proc Natl Acad SciU S A. 2003 Jun 10;100(12):7051-6.
