# Supplementary material for: Hypothesis driven single cell dual oscillator mathematical model of circadian rhythms
Source: PLoS One. 2017 May 9;12(5):e0177197. doi: 10.1371/journal.pone.0177197 (PMC5423656; doi:10.1371/journal.pone.0177197)
Supplement: S3 Table — (DOCX) [file pone.0177197.s013.docx]

**S3 Table. Comparison of previous model predictions on splitting.**

|  | Daan and Berde, 1978 [1] | Oda and Friesen, 2002  [2] | Indic et al., 2008  [3] | Schroder et al., 2012  [4] | New model |
| --- | --- | --- | --- | --- | --- |
| Number of variable /Number of oscillator that coupled | 1/2 | 2/2 | 1/20 | 3/ 200 | 14/2 |
| Nature of model equations | Oscillator is fully defined by series of repeated events in regular intervals | Pittendrigh-Palvidis model  [5] | Kuramoto model  [6] | Goodwin model  [7] | Molecular model that includes essential components of ME oscillator |
| Mechanism behind splitting | Changes in the strength of coupling | Switch in the coupling sign | Due to negative coupling term | Change in the transcription rate due to increased light intensity | Change in the coupling strength and neuropeptide production rate at constant light |

**Reference**

1. Daan S, Berde C. Two coupled oscillators: simulations of the circadian pacemaker in mammalian activity rhythms. J Theor Biol. 1978 Feb 6;70(3):297-313.
2. Oda GA, Friesen WO. A model for “splitting” of running-wheel activity in hamsters. J Biol Rhythms. 2002 Feb;17(1):76-88.
3. Indic P, Schwartz WJ, Paydarfar D. Design principles for phase-splitting behaviour of coupled cellular oscillators: clues from hamsters with ‘split’circadian rhythms. J R Soc Interface. 2008 Aug 6;5(25):873-83.
4. Schroder S, Herzog ED, Kiss IZ. Transcription-based oscillator model for light-induced splitting as antiphase circadian gene expression in the suprachiasmatic nuclei. J Biol Rhythms. 2012 Feb;27(1):79-90.
5. Pittendrigh CS, Kyner WT, Takamura T. The amplitude of circadian oscillations: temperature dependence, latitudinal clines, and the photoperiodic time measurement. J Biol Rhythms. 1991 Dec;6(4):299-313.
6. Kuramoto Y. Chemical oscillations, waves, and turbulence. Berlin, Germany; Springer. 1984. pp. 22–34.
7. Goodwin BC. Oscillatory behavior in enzymatic control processes. **Adv Enzyme Regul**. 1965 Jan 1;3:425-438.
