# Supplementary material for: Hypothesis driven single cell dual oscillator mathematical model of circadian rhythms
Source: PLoS One. 2017 May 9;12(5):e0177197. doi: 10.1371/journal.pone.0177197 (PMC5423656; doi:10.1371/journal.pone.0177197)
Supplement: S5 Table — (DOCX) [file pone.0177197.s015.docx]

**S5 Table. Comparison of ME oscillator in mammals and drosophila**

| Properties | Mammals in SCN | Drosophila | Present mathematical model for ME oscillator hypothesis for mammals in SCN | Ref |
| --- | --- | --- | --- | --- |
| Number of neurons | 20000 clock neurons | 150 clock neurons in brain | Single cell model is constructed to illustrate ME oscillator/ coupling of two single cell ME oscillator is used to explain splitting patterns coupled by neurotransmitter AVP and VIP. | [1, 2] |
| Anatomical locations of ME cells | M and E cells exist in the SCN, but no clear evidence for the exact division of the area that contain M or E cells. The cells are distributed in the caudal, rostral and midrostral part of SCN. | M oscillator is present in small ventral lateral region (s-LNv and half in LN_d_). E oscillator is present in 5^th^ dorsal lateral region (s-LN_v_ and other LN_d_). Even though LN_d_ is heterogeneous group consists of M and E cells, the cells are tightly clustered. | In SCN, M oscillator is taken to be present in ventro-lateral regions and E oscillator is taken to be present dorso-medial. | [1- 4] |
| Molecular components responsible for ME oscillator | Redundant genes are present and they are  3 *per* (*per1/2/3)*and 2 *cry* *( cry1/2*) genes. | There are no redundant genes and only  1 *per/Tim/Cry* genes are present | *per1* and *per2* genes and their proteins are considered. *Cry* genes are not considered. | [1- 5] |
| Response of WT to LL | Oscillations are preserved.  Period increases with increase in the light intensity and follows Aschoff's rule. | Arrhythmic when light intensity is greater than 5 lux. | Period increases with increase in the light intensity (Aschoff's rule). | [1- 5] |
| Response of molecular network/activity pattern to different LD cycle  (Entrainment) | In the molecular network*, per1* mRNA follows dawn and *per2* mRNA follows dusk for various LD cycles. It encodes day length variations. | Presently genes that codes for dawn and dusk are not identified. However, the  wheel running activity shows a M peak in the dawn and E peak in the dusk. | *per1* mRNA follows dawn and *per2* mRNA follows dusk for different LD cycles. | [1- 5] |
| Response of M and E oscillator to LL. | As light intensity increases, period decreases in the M oscillator and in E oscillator the period increases. | Only in *Cry* mutants its observed that as light intensity increases, the period in M oscillator decreases and the period in E oscillator increases (Aschoff's rule) | As light intensity increases, the period in M oscillator decreases and the period in E oscillator increases. | [1- 5] |
| Neurotransmitt-ers as coupling agents that may play a role in ME oscillator | Neuropeptides like VIP, AVP, GABA may play role in ME oscillator, but their roles are not clear yet | The neurotranmitters are PDF, NPF, sNPF, Cha, ITP, and here PDF^+^ neurons are responsible for M acitivity and PDF^-^ and DN_1_neurons are responsible for E activity | VIP and AVP are used as neurotransitters that couple ventrolateral and dorsomedial part of SCN. | [1- 5] |
| Splitting pattern | Activity pattern split into two separate bouts at LL | Bimodal activity rhythm is seen for LL at very low light intensity 0.01 lux as well as in LD. | Different Splitting patterns are observed in the coupled oscillator at constant LL for varying strength of neuropeptides AVP and VIP | [1- 6] |

**References**

1. Helfrich-Förster C. Does the morning and evening oscillator model fit better for flies or mice?. J Biol Rhythms. 2009 Aug 1;24(4):259-70.
2. Kalsbeek A, Merrow M, Roenneberg T, Foster R. Two clocks in the brain: an update of the morning and evening oscillator model in Drosophila. The Neurobiology of Circadian Timing. 2012 Dec 6;199:5.
3. Stoleru D, Peng Y, Agosto J, Rosbash M. Coupled oscillators control morning and evening locomotor behaviour of Drosophila. Nature. 2004 Oct 14;431(7010):862-8.
4. Grima B, Chélot E, Xia R, Rouyer F. Morning and evening peaks of activity rely on different clock neurons of the Drosophila brain. Nature. 2004 Oct 14;431(7010):869-73.
5. Steinlechner S, Jacobmeier B, Scherbarth F, Dernbach H, Kruse F, Albrecht U. Robust circadian rhythmicity of Per1 and Per2 mutant mice in constant light, and dynamics of Per1 and Per2 gene expression under long and short photoperiods. J Biol Rhythms. 2002 Jun;17(3):202-9.
6. Pittendrigh CS. Circadian rhythms, space research and manned space flight. Life SCi Space Res. 1966 Dec;5:122-34.
