# Supplementary material for: Hypothesis driven single cell dual oscillator mathematical model of circadian rhythms
Source: PLoS One. 2017 May 9;12(5):e0177197. doi: 10.1371/journal.pone.0177197 (PMC5423656; doi:10.1371/journal.pone.0177197)
Supplement: S1 Text — (DOCX) [file pone.0177197.s016.docx]

## **S1 Text. Parameter estimation**

Parameter estimation for our model is performed using genetic algorithm (GA) in MATLAB which repeatedly modifies the population of an individual solution [1]. At each step, genetic algorithm randomly select 2 of the parent parameter sets and they are randomly crossed and mutated to produce a new parameter set. Each parameter set was assigned a cost function based on certain criteria that are described below. Over successive generations, the parameter set evolves toward the best solution (Low value of cost function).

*Cost function*

For selecting the best solution with minimum error, the algorithm evaluates a cost function (fitness function) which determines the magnitude of the prediction error. In this work the cost function $C_{1}\left( x\left( t,p \right) \right) or C2(x\left( t,p \right))$ is defined by the squares of the error between the outputs from the model and the experimental data. Using this cost function we reproduced the experimentally observed circadian oscillations. Constraints for the parameter estimation in the order of their relevance were:

(1) Cost function $C_{1}\left( x\left( t,p \right) \right)$ for free running period of oscillation close to 23.7hr

$$C_{1}\left( x\left( t,p \right) \right)={(23.7-\tau_{DD})}^{2}$$

where *x* is the solution of the differential equations 1-13 in the main text , *p* is the parameter vector and $\tau_{DD}$ is the free running period of the model.

(2) Cost function $C_{2}\left( x\left( t,p \right) \right)$for exact peaking time of the molecular components of the circadian clock. $C_{2}\left( x\left( t,p \right) \right)=\sum_{i=1}^{n} \left( \frac{T_{pi}}{24}-\frac{t_{pi}}{\tau_{DD}} \right)^{2}$

Where

$t_{pi}$ is the peaking time of the variable *x_i_* and $T_{pi}$ is the experimentally observed peaking time range of available molecular components.

(3) Cost function $C_{3}\left( x\left( t,p \right) \right)$for the correct phase relationship between the molecular components of the circadian clock.

$$C_{3}\left( x\left( t,p \right) \right)=\sum_{ij} \left( \frac{T_{ij}}{24}-\frac{t_{ij}}{\tau_{DD}} \right)^{2}$$

Where

*t_ij_* is the phase difference between the variable *x_i_* and *x_j_*, *T_ij_* is the experimentally observed phase difference range of available molecular components.

(4) Cost function $C_{4}\left( x\left( t,p \right) \right)$for decent trough/peak ratios of the molecular components.

$$C_{4}\left( x\left( t,p \right) \right)=\sum_{i} \left( z_{i}-\frac{min(x_{i})}{max(x_{i})} \right)^{2}$$

Where

*z_i_* is the experimentally observed trough/peak ratio range of available molecular components.

Thus, the final cumulative cost function is $C\left( x\left( t,p \right) \right)=C_{1}+C_{2}+C_{3}+C_{4}$

**Reference**

1. Yao L, Sethares WA. Nonlinear parameter estimation via the genetic algorithm. IEEE Trans Signal Process. 1994 Apr;42(4):927-35.
