# Supplementary material for: Hypothesis driven single cell dual oscillator mathematical model of circadian rhythms
Source: PLoS One. 2017 May 9;12(5):e0177197. doi: 10.1371/journal.pone.0177197 (PMC5423656; doi:10.1371/journal.pone.0177197)
Supplement: S2 Text — (DOCX) [file pone.0177197.s017.docx]

**S2 Text. modified gating variable for dead zone in PRC**

In mammals, dead zone is seen in experimental PRC during subjective day [1]. To capture the dead zone in our model PRC, we introduced a second gating variable as described below.

$${G=\left( M_{B normalized} \right)}^{10}M_{B max}$$

where

$$M_{B normalized}=\frac{M_{B}-M_{B min}}{M_{B max}-M_{B min}}$$

and *M_B max_* is the maximum value of *M_B_* and *M_B min_* is the minimum value of *M_B_*. Simulated PRC with the new gating variables are shown in the S2 Fig.

**Reference**

1. Pendergast JS, Friday RC, Yamazaki S. Photic entrainment of period mutant mice is predicted from their phase response curves. J Neurosci . 2010 Sep 8;30(36):12179-84.
