# Supplementary material for: Hypothesis driven single cell dual oscillator mathematical model of circadian rhythms
Source: PLoS One. 2017 May 9;12(5):e0177197. doi: 10.1371/journal.pone.0177197 (PMC5423656; doi:10.1371/journal.pone.0177197)
Supplement: S3 Text — (DOCX) [file pone.0177197.s018.docx]

**S3 Text. Period sensitivity**

The period sensitivity is the phase accumulated around steady state orbit after one cycle of revolution and its given as.

$$\frac{\partial\tau}{\partial p_{j}}=\frac{\partial\theta(t,\tau)}{\partial p_{j}}-\frac{\partial\theta(t)}{\partial p_{j}}$$

*t* should be sufficiently large to avoid the transient effect. where τ is the period of the oscillation, *p_j_* is the parameter $\frac{\partial\theta(t)}{\partial p_{j}}$ is the phase response with respect to variation in parameter at time *t,*$\frac{\partial\theta(t,\tau)}{\partial p_{j}}$ is the phase response with respect to variation in parameter after one cycle [1]. The period sensitivity indicates the parameter associated with *per2* loop is more sensitive and this is also seen in various mammalian circadian models [2, 3].

**References**

1. Kramer MA, Rabitz H, Calo JM. Sensitivity analysis of oscillatory systems. Appl Math Model. 1984 Oct 1;8(5):328-40.
2. Forger DB, Peskin CS. A detailed predictive model of the mammalian circadian clock. Proc Natl Acad.Sci U S A. 2003 Dec 9;100(25):14806-11.
3. Wilkins AK, Barton PI, Tidor B. The Per2 negative feedback loop sets the period in the mammalian circadian clock mechanism. PLoS Comput Biol. 2007 Dec 14;3(12):e242.
