# Supplementary material for: Hypothesis driven single cell dual oscillator mathematical model of circadian rhythms
Source: PLoS One. 2017 May 9;12(5):e0177197. doi: 10.1371/journal.pone.0177197 (PMC5423656; doi:10.1371/journal.pone.0177197)
Supplement: S4 Text — (DOCX) [file pone.0177197.s019.docx]

**S4 Text. Coupled oscillator model for morning and evening oscillators**

**M oscillator**

$$\frac{{dM}_{p1m}}{dt}=v_{s1m}\frac{B_{N}^{mm}}{k_{a1m}^{mm}+B_{Nm}^{mm}}-v_{1m}\frac{M_{p1m}}{k_{e1m}+M_{p1m}}-k_{d1m}M_{p1m}+L+v_{cm1}\frac{AVP}{k_{cm}+AVP} (1)$$

$$\frac{{dP}_{1cm}}{dt}=k_{1m}M_{p1m}-v_{2m}\frac{P_{1cm}}{k_{e2m}+P_{1cm}}-k_{d2m}P_{1cm} (2)$$

$$\frac{{dP}_{1nm}}{dt}=k_{2m}P_{1cm}-v_{3m}\frac{P_{1nm}}{k_{e3m}+P_{1nm}}+k_{p1m}-k_{d3}P_{1n} (3)$$

$$\frac{{dM}_{p2m}}{dt}=v_{s2m}\frac{B_{Nm}^{mm}}{k_{a2m}^{mm}+B_{Nm}^{mm}}-v_{4m}\frac{M_{p2m}}{k_{e4m}+M_{p2m}}-k_{d4m}M_{p2m}+L +v_{cm2}\frac{AVP}{k_{cm}+AVP} (4)$$

$$\frac{{dP}_{2cm}}{dt}=k_{3m}M_{p2m}-v_{5m}\frac{P_{2cm}}{k_{e5m}+P_{2cm}}-k_{d5m}P_{2cm} (5)$$

$$\frac{{dP}_{2nm}}{dt}=k_{4m}P_{2cm}-v_{6m}\frac{P_{2nm}}{k_{e6m}+P_{2nm}}+k_{p3m}{PB}_{2m}-k_{p4m}P_{2nm}B_{Nm}-k_{d6m}P_{2nm} (6)$$

$$\frac{{dM}_{Bm}}{dt}=v_{s3m}\frac{k_{I1m}^{2}}{k_{I1m}^{2}+{R_{m}}^{2}+(\frac{R_{m}}{k_{xm}})}+v_{s4m}\frac{P_{2nm}^{wm}}{k_{a3m}^{wm}+P_{2nm}^{wm}}-v_{7m}\frac{M_{Bm}}{k_{e7m}+M_{Bm}}-k_{d7m}M_{Bm} (7)$$

$$\frac{{dB}_{cm}}{dt}=k_{5m}M_{Bm}-v_{8m}\frac{B_{cm}}{k_{e8m}+B_{cm}}-k_{d8m}B_{cm} (8)$$

$$\frac{{dB}_{Nm}}{dt}=k_{6m}B_{cm}-v_{9m}\frac{B_{Nm}}{k_{e9m}+B_{Nm}}+k_{p1m}{PB}_{1m}-k_{p2m}P_{1nm}B_{Nm}+k_{p3m}{PB}_{2m}-k_{p4m}P_{2nm}B_{Nm}-k_{d9m}B_{Nm} (9)$$

$$\frac{{dM}_{Rm}}{dt}=v_{s5m}\frac{B_{Nm}^{sm}}{k_{a4m}^{sm}+B_{Nm}^{sm}}-v_{10m}\frac{M_{Rm}}{k_{e10m}+M_{Rm}}-k_{d10m}M_{Rm} (10)$$

$$\frac{{dR}_{m}}{dt}=k_{7m}M_{Rm}-v_{11m}\frac{R_{m}}{k_{e11m}+R_{m}}-k_{d11m}R_{m} (11)$$

$$\frac{{dPB}_{1m}}{dt}=-k_{p1m}{PB}_{1m}+k_{p2m}P_{1nm}B_{Nm}-k_{d12m}{PB}_{1m} (12)$$

$$\frac{{dPB}_{2m}}{dt}=-k_{p3m}{PB}_{2m}+k_{p4m}P_{2nm}B_{Nm}-k_{d13m}{PB}_{2m} (13)$$

**E oscillator**

$$\frac{{dM}_{p1e}}{dt}=v_{s1e}\frac{B_{Ne}^{me}}{k_{a1e}^{me}+B_{Ne}^{me}}-v_{1}\frac{M_{p1e}}{k_{e1e}+M_{p1e}}-k_{d1e}M_{p1e}+\frac{\beta+VIP \left( v_{ce1}+L \right)}{k_{ce}+VIP} (14)$$

$$\frac{{dP}_{1ce}}{dt}=k_{1e}M_{p1e}-v_{2e}\frac{P_{1ce}}{k_{e2e}+P_{1ce}}-k_{d2e}P_{1ce} (15)$$

$$\frac{{dP}_{1ne}}{dt}=k_{2e}P_{1ce}-v_{3e}\frac{P_{1ne}}{k_{e3e}+P_{1ne}}+k_{p1e}{PB}_{1e}-k_{p2e}P_{1ne}B_{Ne}-k_{d3e}P_{1ne} (16)$$

$$\frac{{dM}_{p2e}}{dt}=v_{s2e}\frac{B_{Ne}^{me}}{k_{a2e}^{me}+B_{Ne}^{me}}-v_{4e}\frac{M_{p2e}}{k_{e4e}+M_{p2e}}-k_{d4e}M_{p2e}+\frac{\beta+VIP \left( v_{ce2}+L \right)}{k_{ce}+VIP} (17)$$

$$\frac{{dP}_{2ce}}{dt}=k_{3e}M_{p2e}-v_{5e}\frac{P_{2ce}}{k_{e5e}+P_{2ce}}-k_{d5e}P_{2ce} (18)$$

$$\frac{{dP}_{2ne}}{dt}=k_{4e}P_{2ce}-v_{6e}\frac{P_{2ne}}{k_{e6e}+P_{2ne}}+k_{p3e}{PB}_{2e}-k_{p4e}P_{2ne}B_{Ne}-k_{d6e}P_{2ne} (19)$$

$$\frac{{dM}_{Be}}{dt}=v_{s3e}\frac{k_{I1e}^{2}}{k_{I1e}^{2}+{R_{e}}^{2}+(\frac{R_{e}}{k_{xe}})}+v_{s4e}\frac{P_{2ne}^{we}}{k_{a3e}^{we}+P_{2ne}^{we}}-v_{7e}\frac{M_{Be}}{k_{e7e}+M_{Be}}-k_{d7e}M_{Be} (20)$$

$$\frac{{dB}_{ce}}{dt}=k_{5e}M_{Be}-v_{8e}\frac{B_{ce}}{k_{e8e}+B_{ce}}-k_{d8e}B_{ce} (21)$$

$$\frac{{dB}_{Ne}}{dt}=k_{6e}B_{ce}-v_{9e}\frac{B_{Ne}}{k_{e9e}+B_{Ne}}+k_{p1e}{PB}_{1e}-k_{p2e}P_{1ne}B_{Ne}+k_{p3e}{PB}_{2e}-k_{p4e}P_{2ne}B_{Ne}-k_{d9e}B_{Ne} (22)$$

$$\frac{{dM}_{Re}}{dt}=v_{s5e}\frac{B_{Ne}^{se}}{k_{a4e}^{se}+B_{Ne}^{se}}-v_{10e}\frac{M_{Re}}{k_{e10e}+M_{Re}}-k_{d10e}M_{Re} (23)$$

$$\frac{{dR}_{e}}{dt}=k_{7e}M_{Re}-v_{11e}\frac{R_{e}}{k_{e11e}+R_{e}}-k_{d11e}R_{m} (24)$$

$$\frac{{dPB}_{1m}}{dt}=-k_{p1m}{PB}_{1m}+k_{p2m}P_{1nm}B_{Nm}-k_{d12m}{PB}_{1m} (25)$$

$$\frac{{dPB}_{2m}}{dt}=-k_{p3m}{PB}_{2m}+k_{p4m}P_{2nm}B_{Nm}-k_{d13m}{PB}_{2m} (26)$$

**AVP and VIP**

$$\frac{dVIP}{dt}=\left\{ \begin{aligned} 0, under DD \\ k_{vs1}\left( P_{1nm}+P_{2nm} \right)-v_{v1}\frac{VIP}{k_{ev1}+VIP}-k_{dv1} VIP , under LL and LD \end{aligned} \right. (27)$$

$$\frac{dAVP}{dt}=k_{vs2}\left( P_{1ne}+P_{2ne} \right)-v_{v2}\frac{AVP}{k_{ev2}+AVP}-k_{dv2} AVP (28)$$
